# Interaction of atomic systems with quantum vacuum beyond electric dipole approximation

**DOI:** 10.1038/s41598-020-62629-0

**Published:** 2020-04-03

**Authors:** Miriam Kosik, Oleksandr Burlayenko, Carsten Rockstuhl, Ivan Fernandez-Corbaton, Karolina Słowik

**Affiliations:** 1 Institute of Physics, Faculty of Physics, Astronomy and Informatics, Nicolaus Copernicus University in Toruń, Grudziadzka 5, 87-100 Torun, Poland; 2 Department of Physics and Technology, V.N. Karazin Kharkiv National University, Kharkiv, Ukraine; 3 Institute of Theoretical Solid State Physics, Karlsruhe Institute of Technology, 76131 Karlsruhe, Germany; 4 Institute of Nanotechnology, Karlsruhe Institute of Technology, 76021 Karlsruhe, Germany

**Keywords:** Atom optics, Nanophotonics and plasmonics, Quantum optics

## Abstract

The photonic environment can significantly influence emission properties and interactions among atomic systems. In such scenarios, frequently the electric dipole approximation is assumed that is justified as long as the spatial extent of the atomic system is negligible compared to the spatial variations of the field. While this holds true for many canonical systems, it ceases to be applicable for more contemporary nanophotonic structures. To go beyond the electric dipole approximation, we propose and develop in this article an analytical framework to describe the impact of the photonic environment on emission and interaction properties of atomic systems beyond the electric dipole approximation. Particularly, we retain explicitly magnetic dipolar and electric quadrupolar contributions to the light-matter interactions. We exploit a field quantization scheme based on electromagnetic Green’s tensors, suited for dispersive materials. We obtain expressions for spontaneous emission rate, Lamb shift, multipole-multipole shift and superradiance rate, all being modified with dispersive environment. The considered influence could be substantial for suitably tailored nanostructured photonic environments, as demonstrated exemplarily.

## Introduction

An excited atomic system, e.g. an atom, a molecule, a quantum dot, can decay radiatively from an excited to a ground state while releasing its energy into the photonic environment. The rate of this process depends on the properties of the environment, and in a pioneering work by Purcell the possibility to control the spontaneous emission lifetimes of atomic systems by tailoring their surroundings was first investigated^1^. Hence, it has also been called the Purcell effect. The effect has been experimentally verified in various types of cavities or band-gap environments, including semiconductor microstructures^2^, photonic crystals^3^, and plasmonic nanoparticles, where the emission rate was enhanced up to three orders of magnitude^4,5^. Besides emission enhancement on its own, photonic environment equally affects the interactions between multiple atomic systems. In vacuum, examples such as dipole-dipole coupling^6^, or collective phenomena like Dicke superradiance have been explored^7,8^. Also, all these effects can be tailored by suitably engineering the photonic environment^9,10^.

The influence of photonic environment on these phenomena is usually quantified in the electric dipole approximation. This is justified when the electric field shows a negligible spatial variation across the size of the atomic system. Steps beyond may be required if the atomic system is less than an order of magnitude smaller than the wavelength of light it is coupled to, as demonstrated in semiconductor quantum dots^11^ or in Rydberg excitons^12^. Similarly, corrections beyond the electric dipole approximation may be necessary if the electromagnetic field is focused into spots comparable in size to the atomic system. The latter can be realized by nanoscopic environments and picocavities, capable to localize the electric field into nanometric spatial domains, providing high intensities and spatial modulations at the length scale of tens of nanometers. This tends to be comparable to the size scale of molecules or quantum dots^13–15^. Then the usual mismatch of size scales of photonic modes and atomic systems is reduced, which leads to enhanced interaction probability if the photonic modes and the atomic system overlap in space. The resulting need to include corrections beyond the electric dipole approximation to properly quantify light-matter coupling was demonstrated for quantum dots near plasmonic nanoparticles^16^.

Furthermore, and potentially more interestingly, nanostructured environments may also open light-matter interaction channels beyond that one corresponding to coupling of electric field with electric dipoles. For example, light concentration in nanoscopic regions causes modulations of electromagnetic field at spatial distances comparable to the size of atomic systems, which may couple to electric-quadrupolar or higher-order moments. In addition, due to their large refractive index, dielectric nanomaterials offer the possibility of strong concentration of magnetic fields^17^. This prompts to consider not just electric-multipolar contributions but at the same time their magnetic counterparts. Until now, enhancement of the rate of a magnetic dipole emission by a nanostructure was considered^18^ and reported experimentally in lanthanide ions^19–22^. Large enhancement of quadrupole^23–25^ and even higher-order transitions was equally predicted. Transitions driven with several multipolar mechanisms have been considered^26^ and observed^16,27^ respectively in semiconductor quantum dots and transition-metal/lanthanide ions. Recently, it was pointed out that the simultaneous existence of several interaction channels enhanced in intensity with photonic nanostructures might open new avenues on the route to control spontaneous emission, related to their controlled interference^28^. To account for these effects, treatments beyond the point-dipole approximation were developed^29–31^, based on spatial integration of the transition elements expressed in terms of wavefunctions of the atomic system. Nonclassical corrections to these transition rates originating from effects such as nonlocality, electronic spill-out and Landau damping have been considered in ref. ^32^. Although such approaches take into account all multipolar orders of light-matter interaction, they require information on the atomic system’s wavefunctions, which are in general complex multidimentional structures. They are hardly accessible experimentally and their calculation may be a significant computational effort already in case of few-electron systems.

In this work, we develop an analytic theory based on the multipolar coupling Hamiltonian, in which the spatial extent of the atomic system is taken into account in terms of multipolar transition moments rather than wavefunctions. These moments can be evaluated with smaller computational effort than full wavefunctions, found in literature^33,34^ or measured^21^,^35^. Contrary to the previous works mentioned above, we evaluate not just transition rates, but also energy shifts. In the case of an individual atomic system we evaluate Purcell enhancement of spontaneous emission and Lamb-shift modification in nanostructured dispersive environment. Additionally, we provide expressions to evaluate interaction strengths and collective emission rates of multiple atomic systems immersed in the same environment, while considering the magnetic-dipolar and electric-quadrupolar interaction channels. Our method exploits field quantization in dispersive media^36^, in which the optical properties of the environment are expressed in terms of the electromagnetic Green’s tensor, defined by the spatial and spectral dependence of the electric permittivity. Our result is an extension to those results previously obtained in electric-dipole approximation in refs. ^9,37^. Here, we include two next-order terms of the multipolar coupling Hamiltonian^38^, namely the magnetic dipole and the electric quadrupole. This unlocks qualitatively new routes to tailor light-matter coupling exploiting interference effects.

The work is organized as follows: in the next section we introduce the general framework used to describe light-matter coupling in the presence of dispersive and absorptive structured materials beyond electric-dipole approximation. Examples of application of the theory to specific geometries are given at the end of the Results section. Detailed numerical results and lengthy calculations are provided in the Supplementary Information available online.

## Results

In this section, we introduce the general framework used to describe light-matter coupling in the presence of dispersive and absorptive structured materials beyond electric-dipole approximation. After giving a statement of the problem, we offer expressions for spontaneous emission rate and Lamb shift of a single atomic system in such environment at first. Next, we generalize these expressions to many-atom systems. Finally, examples of application of the theory to specific geometries are given. Lengthy calculations are documented in Supplementary Information.

### Atomic system

We assume that the atomic system can be approximated by two active energy levels, separated by energy $\hslash {\omega }_{0}$, where $\hslash$ stands for the reduced Planck’s constant. The corresponding ground and excited states are denoted by $\left|g\right\rangle$ and $\left|e\right\rangle$, respectively. The system is fully described by a set of Pauli operators: the lowering operator $\sigma =\left|g\right\rangle \left\langle e\right|$ and the inversion operator ${\sigma }_{z}=\left|e\right\rangle \left\langle e\right|-\left|g\right\rangle \left\langle g\right|$, following the usual commutation rules $[\sigma ,{\sigma }^{\dagger }]=-{\sigma }_{z}$, $[\sigma ,{\sigma }_{z}]=2\sigma$. The free Hamiltonian of the system reads ${{\mathscr{H}}}_{0}=\hslash {\omega }_{0}{\sigma }^{\dagger }\sigma$. This Hamiltonian can be generalized to the case of multiple emitters in a straightforward manner, as done in one of the following subsections.

### Quantized electromagnetic field in dispersive media

In this work we follow the quantization scheme in dispersive and absorbing media, developed in refs. ^39–42^. We restrict ourselves to nonmagnetic matter with relative permeability $\mu =1$. The constitutive equation relating the temporal Fourier components of the displacement field ${\bf{D}}({\bf{r}},\omega )$, the electric field ${\bf{E}}({\bf{r}},\omega )$, and medium polarization ${\bf{P}}({\bf{r}},\omega )$ in an absorbing medium takes the form ${\bf{D}}({\bf{r}},\omega )={\varepsilon }_{0}{\bf{E}}({\bf{r}},\omega )+{\bf{P}}({\bf{r}},\omega )={\varepsilon }_{0}\varepsilon ({\bf{r}},\omega ){\bf{E}}({\bf{r}},\omega )+{{\bf{P}}}_{N}({\bf{r}},\omega )$. Here, ${{\bf{P}}}_{N}({\bf{r}},\omega )$ describes a noise contribution to the polarization ${\bf{P}}({\bf{r}},\omega )$ arising from vacuum fluctuations in an absorbing medium. The related noise current density reads ${{\bf{j}}}_{N}({\bf{r}},\omega )=-i\omega {{\bf{P}}}_{N}({\bf{r}},\omega )$.

Since we investigate vacuum-induced effects, we are interested in the case of a vanishing mean electric field. Then, the only field is related to noise current fluctuations and can be expressed as

1 $${\bf{E}}\left({\bf{r}},\omega \right)=i{\mu }_{0}\omega \int {d}^{3}r{\prime} {\bf{G}}\left({\bf{r}},{\bf{r}}{\prime} ,\omega \right){{\bf{j}}}_{N}\left({\bf{r}}{\prime} ,\omega \right),$$

where ${\mu }_{0}$ stands for vacuum permeability.

The dyadic tensor ${\bf{G}}\left({\bf{r}},{\bf{r}}{\prime} ,\omega \right)$ is the full electromagnetic Green’s tensor characterizing the environment, determined from the Maxwell-Helmholtz equation

2 $$\left[\nabla \times \nabla \times -\frac{{\omega }^{2}}{{c}^{2}}{\boldsymbol{\varepsilon }}\left({\bf{r}},\omega \right)\right]{\bf{G}}\left({\bf{r}},{\bf{r}}{\prime} ,\omega \right)=I\delta \left({\bf{r}}-{\bf{r}}{\prime} \right),$$

with $I$ representing the unit dyadic. The boundary condition for the Green’s tensor at infinity reads ${\bf{G}}\left({\bf{r}},{\bf{r}}{\prime} ,\omega \right)\to 0$ for $\left|{\bf{r}}-{\bf{r}}{\prime} \right|\to \infty$. The Green’s tensor serves as the kernel that connects the electric field ${\bf{E}}\left({\bf{r}},\omega \right)$ with its source at position ${\bf{r}}{\prime}$.

The requirement for the canonical equal-time commutation relations of quantized fields to hold allows one to express the noise current in the form^39–41^

3 $${{\bf{j}}}_{N}({\bf{r}},\omega )=\omega \sqrt{\frac{\hslash {\varepsilon }_{0}}{\pi }{\rm{Im}}{\boldsymbol{\varepsilon }}({\bf{r}},\omega )}\ {{\bf{f}}}_{\omega }\left({\bf{r}}\right).$$

Here, ${{\boldsymbol{\varepsilon }}}_{0}$ represents the electric permittivity of vacuum and ${\boldsymbol{\varepsilon }}\left({\bf{r}},\omega \right)={\rm{Re}}\ {\boldsymbol{\varepsilon }}\left({\bf{r}},\omega \right)+i\ {\rm{Im}}\ {\boldsymbol{\varepsilon }}\left({\bf{r}},\omega \right)$ is the relative permittivity of the dispersive and absorptive medium surrounding the atomic system. For simplicity, we assume isotropic media, so that the permittivity can be expressed as a scalar function. The bosonic operator fields take the form ${{\bf{f}}}_{\omega }\left({\bf{r}}\right)={\sum }_{{\rm{j}}}\,{f}_{\omega ,{\rm{j}}}\left({\bf{r}}\right){{\bf{e}}}_{{\rm{j}}}$, where ${\rm{j}}\in \{x,y,z\}$ and ${{\bf{e}}}_{{\rm{j}}}$ is a unit vector in the ${{\rm{j}}}^{{\rm{th}}}$ direction. They obey the following commutation relations^39–41^

4 $$\begin{array}{ccc}\left[{f}_{\omega ,{\rm{j}}}\left({\bf{r}}\right),{f}_{\omega {\prime} ,{\rm{k}}}\left({\bf{r}}{\prime} \right)\right]=0, &  & \\ \left[{f}_{\omega ,{\rm{j}}}\left({\bf{r}}\right),{f}_{\omega {\prime} ,{\rm{k}}}^{\dagger }\left({\bf{r}}{\prime} \right)\right]={\delta }_{{\rm{jk}}}\delta (\omega -\omega {\prime} )\delta ({\bf{r}}-{\bf{r}}{\prime} ). &  & \end{array}$$

Finally, the electric field can be expressed in terms of bosonic operators as follows

5 $${\bf{E}}\left({\bf{r}},\omega \right)=i\sqrt{\frac{\hslash }{\pi {{\boldsymbol{\varepsilon }}}_{0}}}\frac{{\omega }^{2}}{{c}^{2}}\int {d}^{3}{r}^{{\prime} }\sqrt{{\rm{Im}}\ {\boldsymbol{\varepsilon }}\left({\bf{r}}{\prime} ,\omega \right)}\ {\bf{G}}\left({\bf{r}},{\bf{r}}{\prime} ,\omega \right){{\bf{f}}}_{\omega }\left({\bf{r}}{\prime} \right),$$

where $c$ is the vacuum speed of light.

In the Schrödinger picture, the positive frequency part ${{\bf{E}}}^{(+)}\left({\bf{r}}\right)={\left[{{\bf{E}}}^{(-)}\left({\bf{r}}\right)\right]}^{\dagger }$ of the electric field operator ${\bf{E}}\left({\bf{r}}\right)={{\bf{E}}}^{(+)}\left({\bf{r}}\right)+{{\bf{E}}}^{(-)}\left({\bf{r}}\right)$ is obtained through the integration ${{\bf{E}}}^{(+)}\left({\bf{r}}\right)={\int }_{0}^{\infty }d\omega \ {\bf{E}}\left({\bf{r}},\omega \right)$. Similarly, the positive frequency part of the magnetic field is expressed given by ${{\bf{B}}}^{(+)}\left({\bf{r}}\right)={\int }_{0}^{\infty }d\omega \ {\bf{B}}\left({\bf{r}},\omega \right)$, where the frequency components of the magnetic fields are connected to the corresponding electric field through ${\bf{B}}\left({\bf{r}},\omega \right)=-\frac{i}{\omega }\nabla \times {\bf{E}}\left({\bf{r}},\omega \right)$.

The free-field Hamiltonian reads ${{\mathscr{H}}}_{{\rm{field}}}=\int {d}^{3}r\int \ d\omega \ \hslash \omega \ {{\bf{f}}}_{\omega }{\left({\bf{r}}\right)}^{\dagger }{{\bf{f}}}_{\omega }\left({\bf{r}}\right)$.

### Coupling Hamiltonian

The electric dipole approximation is based on the assumption that the electric field can be approximated as constant across the spatial extent of the atomic system. In particular, this means that direct coupling of the emitter with the magnetic field is ignored and spatial modulations of the electric fields are neglected as well. Typically, the above assumption is very well met: the scale of spatial modulations of the electric field is set by its wavelength, usually in the optical or near-infrared range, while the modulations of the field’s envelope are even slower and thus negligible. This assumption holds true in free space or traditional cavities, if the atomic system is represented by an atom or a molecule. However, the assumption might no longer be applicable if the emitter is positioned within a subwavelength electromagnetic hotspot, e.g. in photonic crystal cavities, in close vicinity of plasmonic nanostructures^16^, near picocavities or even in free space when geometrically large emitters like semiconductor quantum dots are considered^11,26,31^. For this reason, we include two higher-order terms of the multipolar coupling Hamiltonian, which include first-order spatial derivatives of the electric field^38^. The Hamiltonian is given in the rotating wave approximation, valid as long as the coupling strengths are small with respect to the transition frequency ${\omega }_{0}$

6 $${{\mathscr{H}}}_{{\rm{int}}}=-\left[{{\bf{E}}}_{0}^{(-)}\cdot {\bf{d}}+{{\bf{B}}}_{0}^{(-)}\cdot {\bf{m}}+\nabla {{\bf{E}}}_{0}^{(-)}:{\bf{Q}}\right]\sigma -{\sigma }^{\dagger }\left[{{\bf{d}}}^{\dagger }\cdot {{\bf{E}}}_{0}^{(+)}+{{\bf{m}}}^{\dagger }\cdot {{\bf{B}}}_{0}^{(+)}+{{\bf{Q}}}^{\dagger }:\nabla {{\bf{E}}}_{0}^{(+)}\right],$$

where the fields are evaluated at the position of the atomic system ${{\bf{r}}}_{0}$, and for brevity we denote ${{\bf{E}}}_{0}^{(\pm )}\equiv {{\bf{E}}}^{(\pm )}({{\bf{r}}}_{0})$. We assume that the electric field derivatives exist at this position, i.e. the atomic system should not be placed directly at an interface between two different media. Above, ${\bf{d}}=\langle g| \widehat{{\bf{d}}}| e\rangle$ and ${\bf{m}}=\langle g| \widehat{{\bf{m}}}| e\rangle$ are the electric and magnetic transition dipole moment elements, and ${\bf{Q}}=\langle g| \widehat{{\bf{Q}}}| e\rangle$ is the electric transition quadrupole moment element, respectively. The dot denotes the standard scalar product of vectors, the double dot product of tensors is defined as ${\bf{C}}:{\bf{D}}\equiv {\sum }_{ij}{C}_{ij}{D}_{ji}$, while $\nabla {{\bf{E}}}^{(\pm )}$ is a dyadic product. In the case of a real electric quadrupole moment tensor, the quadrupolar contribution to the coupling Hamiltonian can be equivalently rewritten as ${{\bf{Q}}}^{\dagger }:\nabla {{\bf{E}}}^{(+)}\left({\bf{r}}\right)=\left({{\bf{Q}}}^{\dagger }\,\nabla \right)\cdot {{\bf{E}}}^{(+)}\left({\bf{r}}\right)={\sum }_{ij}{Q}_{ij}^{\ast }{\partial }_{j}{E}_{i}^{(+)}\left({\bf{r}}\right)$. Please note that different degrees of freedom in the operators above are denoted as follows:

- The degree of freedom related to the two-dimensional Hilbert space spanned by $\{\left|g\right\rangle ,\left|e\right\rangle \}$ is already included in the symbols of transition moments, and is relevant for Hermitian conjugation, e.g. ${{\bf{Q}}}^{\dagger }={\left(\left\langle g\right|\widehat{{\bf{Q}}}\left|e\right\rangle \right)}^{\dagger }=\left\langle e\right|\widehat{{\bf{Q}}}\left|g\right\rangle$; permanent multipole moments are assumed negligible, e.g. $\left\langle g\right|\widehat{{\bf{Q}}}\left|g\right\rangle =\left\langle e\right|\widehat{{\bf{Q}}}\left|e\right\rangle =0$,
- the dipole moment vectors and the quadrupole moment tensor have elements corresponding to the $x,y,z$ spatial directions, e.g. ${d}_{i}$, ${Q}_{ij}$, responsible for the orientation of the multipolar moment,
- finally, the fields depend on position in space ${\bf{r}}$, and each element of the Green’s tensor is a function of the observation point ${\bf{r}}$ and the source point ${\bf{r}}{\prime}$.

### Emission properties of single atomic system

To derive the spontaneous emission rate of an atomic system, we proceed as follows: First, Heisenberg equations are found for the atomic and the field operators. The equations for the field are then formally integrated, so that the field operators are expressed through the atomic ones. The result is then inserted into atomic equations, so that field variables are completely eliminated from the description. The resulting complicated integro-differential equations are simplified in the Markovian approximation, where the memory effects are neglected. As a result, we obtain effective dynamics of the atomic system alone. The procedure is a generalization of the one introduced in ref. ^9^, where only the electric dipole interaction term was taken into account. Since the equations tend to be lengthy, we describe the consecutive steps in detail in Section 1 of the Supplementary Information. The effective evolution of the atomic system reads

7 $$\begin{array}{lll}\dot{\sigma } & = & -\left[\frac{\gamma }{2}+i\left({\omega }_{0}+\delta \right)\right]\sigma -\frac{i}{\hslash }{\sigma }_{z}\left[{{\bf{d}}}^{\dagger }\cdot {{\bf{E}}}_{0,{\rm{free}}}^{(+)}+{{\bf{m}}}^{\dagger }\cdot {{\bf{B}}}_{0,{\rm{free}}}^{(+)}+{{\bf{Q}}}^{\dagger }:\nabla {{\bf{E}}}_{0,{\rm{free}}}^{(+)}\right]\end{array}$$

8 $$\begin{array}{lll}{\dot{\sigma }}_{z} & = & -\gamma \left({\sigma }_{z}+{\mathbb{1}}\right)+\frac{2i}{\hslash }\left\{{\sigma }^{\dagger }\left[{{\bf{d}}}^{\dagger }\cdot {{\bf{E}}}_{0,{\rm{free}}}^{(+)}+{{\bf{m}}}^{\dagger }\cdot {{\bf{B}}}_{0,{\rm{free}}}^{(+)}+{{\bf{Q}}}^{\dagger }:\nabla {{\bf{E}}}_{0,{\rm{free}}}^{(+)}\right]-\left[{{\bf{E}}}_{0,{\rm{free}}}^{(-)}\cdot {\bf{d}}+{{\bf{B}}}_{0,{\rm{free}}}^{(-)}\cdot {\bf{m}}+\nabla {{\bf{E}}}_{0,{\rm{free}}}^{(-)}:{\bf{Q}}\right]\sigma \right\},\end{array}$$

where the fields are always evaluated at ${{\bf{r}}}_{0}$, ${\mathbb{1}}$ is the identity operator in the atomic Hilbert space, $\gamma$ is the spontaneous emission rate, and $\delta$ stands for the analogue of Lamb shift, calculated beyond the electric dipole approximation. Explicit expressions for these quantities are given in the following. The “free” subscript corresponds to free fields, i.e. fields that are not influenced by atomic back-action. In the vacuum state, these fields account for vacuum fluctuations and their mean value vanishes. The influence of the photonic environment is taken into account through the modified Green’s tensor. The emission rate $\gamma$ includes contributions from the electric dipole, magnetic dipole, and electric quadrupole channels, as well as their interference. The rate is expressed as

9 $$\gamma =\frac{2}{\hslash {\varepsilon }_{0}}\frac{{\omega }_{0}^{2}}{{c}^{2}}{\sum }_{mn}{D}_{m}^{r\dagger }{D}_{n}^{r{\prime} }{\rm{Im}}\ {G}_{mn}\left({\bf{r}},{\bf{r}}{\prime} ,{\omega }_{0}\right){| }_{\begin{array}{c}{\bf{r}}={{\bf{r}}}_{0}\end{array}}{\bf{r}}{\prime} ={{\bf{r}}}_{0},$$

where we have defined a “generalized transition moment” ${{\bf{D}}}^{r}$ with components

10 $${D}_{m}^{r}={d}_{m}+\sum _{k}\left({Q}_{mk}+\frac{i}{{\omega }_{0}}{\sum }_{p}{{\boldsymbol{\varepsilon }}}_{pkm}{m}_{p}\right)\frac{\partial }{\partial {r}_{k}},$$

with $m,k,p\in \{x,y,z\}$, ${G}_{mn}^{{}^{{\prime\prime} }}$ denotes the imaginary part of an $mn$ element of the Green’s tensor and ${\varepsilon }_{pkm}$ is the Levi-Civita antisymmetric symbol. The derivatives in Eq. (10) should be evaluated at the position of the atomic system. Please note that the “generalized moment” is in fact a differential operator that acts on the Green’s tensor, i.e. the “moment” combines atomic and field properties. We stress that ${{\bf{D}}}^{r}$ becomes a purely atomic quantity only in the electric dipole approximation. Please note that in this case expression (9) is reduced to the well known form $\gamma =\frac{2}{\hslash {\varepsilon }_{0}}\frac{{\omega }_{0}^{2}}{{c}^{2}}{{\bf{d}}}^{\dagger }\cdot {\rm{Im}}\ {\bf{G}}\left({{\bf{r}}}_{0},{{\bf{r}}}_{0},{\omega }_{0}\right)\cdot {\bf{d}}$^9,37,43^.

The Lamb shift in Eq. (7) is expressed through a principal-value integral:

11 $$\delta =\frac{1}{\hslash \pi {\epsilon }_{0}{c}^{2}}{\mathscr{P}}{\int }_{0}^{\infty }d\omega \frac{{\omega }^{2}}{\omega -{\omega }_{0}}{\sum }_{mn}{D}_{m}^{r\dagger }(\omega ){D}_{n}^{r{\prime} }(\omega )\ {\rm{Im}}\ {G}_{mn}\left({\bf{r}},{\bf{r}}{\prime} ,\omega \right){| }_{\begin{array}{c}{\bf{r}}={{\bf{r}}}_{0},\\ {\bf{r}}{\prime} ={{\bf{r}}}_{0}\end{array}}$$

where

12 $$\,{D}_{m}^{r}\left(\omega \right)={d}_{m}+\sum _{k}\left({Q}_{mk}+\frac{i}{\omega }{\sum }_{p}{{\boldsymbol{\varepsilon }}}_{pkm}{m}_{p}\right)\frac{\partial }{\partial {r}_{k}},$$

and the generalized moment in Eq. (10) is ${D}_{m}^{r}={D}_{m}^{r}\left({\omega }_{0}\right)$. Again, in electric dipole approximation expression (11) reduces to the familiar form derived in refs. ^9,44^.

#### General comments

Before we move to the next section, we will make a few general comments.

The emission and frequency shifts originate from coupling to quantized electromagnetic vacuum and can be derived in the quantum model, albeit their enhancement with respect to free space can be expressed with classical quantities. The enhancement depends on the geometry of the surroundings of the emitter, which determines the properties of the field, here accounted for in terms of the Green’s tensor. The tensor is governed by the classical electric permittivity. Such description does not take into account the electron tunneling effects which might appear between the atomic system and metallic nanoparticles for separations below 1 nm^45^.

The Green’s tensor ${\bf{G}}\left({\bf{r}},{\bf{r}}{\prime} ,\omega \right)={{\bf{G}}}_{h}\left({\bf{r}},{\bf{r}}{\prime} ,\omega \right)+{{\bf{G}}}_{s}\left({\bf{r}},{\bf{r}}{\prime} ,\omega \right)$ can be decomposed into a sum of a homogeneous term ${{\bf{G}}}_{h}$ and a scattered part ${{\bf{G}}}_{s}$^46^. The homogeneous term corresponds to the response in free space or a homogeneous medium, while the scattered one describes influence of scatterers in the environment, e.g. extended interfaces among different media, nanostructured particles, or photonic crystals. The contribution of the homogeneous part of the Green’s tensor is already included in the homogeneous-medium spontaneous emission rate or the respective Lamb shift, while the contribution of the scattered part is of general interest. The scattered contribution is frequently finite at the origin, as ${\bf{r}},{\bf{r}}{\prime} \to {{\bf{r}}}_{0}$.

From an analysis of the generalized multipole moment, we find that the electric dipole component depends on the imaginary part of Green’s tensor, while the electric quadrupole and the magnetic dipole components are proportional to the sum and difference of the corresponding derivatives of the imaginary part of the Green’s function: ${Q}_{km}\left({\partial }_{k}+{\partial }_{m}\right)$, ${m}_{p}\left({\partial }_{k}-{\partial }_{m}\right)$, $p\ne k,m$, respectively. A simple $\frac{\pi }{4}$-rotation of the coordinate system shows that these can be independently tailored, since there are no general restrictions on the ratios of values of a function and its derivatives in different directions at a given point. This observation is a starting point to consider their full interference and engineer environments which might support it^28^.

Generalizing the expressions for the transition rate beyond the electric dipole approximation not only allows to consider corrections to the atomic systems’ dynamics in cavities of extreme geometries. More importantly, it is a tool to describe, e.g. optical activity of chiral molecules for which an interplay between the electric and magnetic dipolar coupling of matter and light, i.e. interference of the two transition mechanisms, plays a crucial role. In centrosymmetric systems, parity is a good quantum number, allowing one to identify transitions either described by the electric dipole mechanism or a combination of electric quadrupole and magnetic dipole. Such systems could be considered sources of photons with well-defined parity, corresponding to a given transition mechanism.

### Emission properties of multiple atomic systems

The same formalism can be applied to the case where multiple two-level atomic systems, indexed with $\alpha$, share the same photonic environment. The systems do not need to be identical, but we assume the separations of their transition frequencies ${\omega }_{\alpha }$ to be small with respect to the scale of spectral modulations of the properties of the photonic environment. This assumption will be relevant for the Markovian approximation. We additionally assume that the systems do not directly interact. However, the shared environment can be a carrier of interatomic coupling, as we will see below.

In the case of multiple atomic systems, the Hamiltonian from Eq. (6) should be generalized to the form

13 $${\mathscr{H}}={{\mathscr{H}}}_{{\rm{field}}}+\sum _{\alpha }{{\mathscr{H}}}_{\alpha }+\sum _{\alpha }{{\mathscr{H}}}_{{\rm{int}},\alpha },$$

where ${{\mathscr{H}}}_{\alpha }=\hslash {\omega }_{\alpha }{\sigma }_{\alpha }^{\dagger }{\sigma }_{\alpha }$, and ${{\mathscr{H}}}_{{\rm{int}},\alpha }$ is given by Eq. (6) with the operator $\sigma$ replaced with ${\sigma }_{\alpha }$ and fields evaluated at positions ${{\bf{r}}}_{\alpha }$ of the ${\alpha }^{{\rm{th}}}$ atomic system ${{\bf{E}}}^{(\pm )}({{\bf{r}}}_{0})\to {{\bf{E}}}_{\alpha }^{(\pm )}\equiv {{\bf{E}}}^{(\pm )}({{\bf{r}}}_{\alpha })$, ${{\bf{B}}}^{(\pm )}({{\bf{r}}}_{0})\to {{\bf{B}}}_{\alpha }^{(\pm )}\equiv {{\bf{B}}}^{(\pm )}({{\bf{r}}}_{\alpha })$. A set of Pauli operators ${\sigma }_{\alpha }$, ${\sigma }_{z,\alpha }$ describes the ${\alpha }^{{\rm{th}}}$ atomic system. Different systems are naturally independent of each other, so the commutation rules for Pauli operators read $\left[{\sigma }_{\alpha },{\sigma }_{\beta }\right]=0$, $\left[{\sigma }_{\alpha },{\sigma }_{\beta }^{\dagger }\right]=-{\sigma }_{z,\alpha }{\delta }_{\alpha \beta }$, $\left[{\sigma }_{\alpha },{\sigma }_{z,\beta }\right]=2{\sigma }_{\alpha }{\delta }_{\alpha \beta }$.

Steps to derive evolution equations of atomic operators in the Markovian approximation are listed in Section 2 of the Supplementary Information. The resulting equations read

14 $$\begin{array}{lll}{\dot{\sigma }}_{\beta } & = & \left[-i\left(\bar{\omega }+{\delta }_{\beta }\right)-{\gamma }_{\beta \beta }\right]{\sigma }_{\beta }+{\sum }_{\alpha \ne \beta }(i{\xi }_{\alpha \beta }+{\gamma }_{\alpha \beta }){\sigma }_{z,\beta }{\sigma }_{\alpha }-\frac{i}{\hslash }{\sigma }_{z,\beta }\left[{{\bf{Q}}}^{\dagger \beta }:\nabla {{\bf{E}}}_{\beta ,{\rm{free}}}^{(+)}+{{\bf{m}}}_{\beta }^{\dagger }\cdot {{\bf{B}}}_{\beta ,{\rm{free}}}^{(+)}+{{\bf{d}}}_{\beta }^{\dagger }\cdot {{\bf{E}}}_{\beta ,{\rm{free}}}^{(+)}\right]\end{array}$$

15 $$\begin{array}{lll}{\dot{\sigma }}_{z,\beta } & = & -{\gamma }_{\beta \beta }\left({\sigma }_{z,\beta }+{\mathbb{1}}\right)+2i\left({\xi }_{\alpha \beta }{\sigma }_{\alpha }^{\dagger }{\sigma }_{\beta }-{\xi }_{\alpha \beta }^{\star }{\sigma }_{\beta }^{\dagger }{\sigma }_{\alpha }\right)\\  &  & +\frac{2i}{\hslash }\left\{{\sigma }_{\beta }^{\dagger }\left[{{\bf{d}}}_{\beta }^{\dagger }\cdot {{\bf{E}}}_{\beta ,{\rm{free}}}^{(+)}+{{\bf{m}}}_{\beta }^{\dagger }\cdot {{\bf{B}}}_{\beta ,{\rm{free}}}^{(+)}+{{\bf{Q}}}_{\beta }^{\dagger }:\nabla {{\bf{E}}}_{\beta ,{\rm{free}}}^{(+)}\right]\right.\\  &  & -\left.\left[{{\bf{E}}}_{\beta ,{\rm{free}}}^{(-)}\cdot {{\bf{d}}}_{\beta }+{{\bf{B}}}_{\beta ,{\rm{free}}}^{(-)}\cdot {{\bf{m}}}_{\beta }+\nabla {{\bf{E}}}_{\beta ,{\rm{free}}}^{(-)}:{{\boldsymbol{Q}}}_{\beta }\right]{\sigma }_{\beta }\right\}.\end{array}$$

For a better understanding, it is useful to note that the same equations can be derived for a collection of atomic systems described by an effective Hamiltonian of the form

16 $${{\mathscr{H}}}_{{\rm{eff}}}={\sum }_{\alpha }\hslash \left(\bar{\omega }+{\delta }_{\alpha }\right){\sigma }_{\alpha }^{\dagger }{\sigma }_{\alpha }+\hslash {\sum }_{\alpha  > \beta }\left({\xi }_{\alpha \beta }{\sigma }_{\alpha }^{\dagger }{\sigma }_{\beta }+{\xi }_{\alpha \beta }^{\star }{\sigma }_{\beta }^{\dagger }{\sigma }_{\alpha }\right).$$

In the Hamiltonian in Eq. (13), the photonic environment explicitly plays the role of the interaction carrier. In (16) the environment is eliminated, and an effective and direct multipole-multipole coupling is present instead with a strength

17 $${\xi }_{\alpha \beta }=\sum _{mn}\left\{{\mathscr{P}}{\int }_{0}^{\infty }d\omega {R}_{mn}^{\alpha \beta }\left(\omega \right)\frac{{\omega }^{2}}{\omega -\bar{\omega }}{\rm{Im}}\ {G}_{mn}\left({\bf{r}}{\prime} ,{\bf{r}},\omega \right){| }_{\begin{array}{c}{\bf{r}}={{\bf{r}}}_{\beta },\\ {\bf{r}}{\prime} ={{\bf{r}}}_{\alpha }\end{array}}+{I}_{mn}^{\alpha \beta }\left(\bar{\omega }\right)\pi {\bar{\omega }}^{2}{\rm{Im}}\ {G}_{mn}\left({\bf{r}}{\prime} ,{\bf{r}},\bar{\omega }\right){| }_{\begin{array}{c}{\bf{r}}={{\bf{r}}}_{\beta }\\ {\bf{r}}{\prime} ={{\bf{r}}}_{\alpha }\end{array}}\right\},$$

where ${R}_{mn}^{\alpha \beta }(\omega )$ and ${I}_{mn}^{\alpha \beta }(\omega )$ are expressed through multipolar elements and differential operators, as given in Section 2 of the Supplementary Information. From Eq. S17 in Section 3 of the Supplementary Information it follows that if the transition frequency is sufficiently large, the expression for the coupling can be simplified to the form

18 $$\begin{array}{ccc}{\xi }_{\alpha \beta } & = & \pi {\bar{\omega }}^{2}\sum _{mn}\left\{{R}_{mn}^{\alpha \beta }(\bar{\omega }){\rm{Re}}\ {G}_{mn}\left({\bf{r}}{\prime} ,{\bf{r}},\bar{\omega }\right){| }_{\begin{array}{c}{\bf{r}}={{\bf{r}}}_{\beta },\\ {\bf{r}}{\prime} ={{\bf{r}}}_{\alpha }\end{array}}+{I}_{mn}^{\alpha \beta }\left(\bar{\omega }\right){\rm{Im}}\ {G}_{mn}\left({\bf{r}}{\prime} ,{\bf{r}},\bar{\omega }\right){| }_{\begin{array}{c}{\bf{r}}={{\bf{r}}}_{\beta },\\ {\bf{r}}{\prime} ={{\bf{r}}}_{\alpha }\end{array}}\right\}\\  &  & +{\int }_{0}^{\infty }\frac{{\omega }^{2}\bar{\omega }}{{\omega }^{2}+{\bar{\omega }}^{2}}{R}_{mn}^{\alpha \beta }(i\omega ){\rm{Re}}\ {G}_{mn}\left({\bf{r}}{\prime} ,{\bf{r}},i\omega \right){| }_{\begin{array}{c}{\bf{r}}={{\bf{r}}}_{\beta },\\ {\bf{r}}{\prime} ={{\bf{r}}}_{\alpha }\end{array}},\end{array}$$

where the principal value integral is no longer present and the integration is now performed along the imaginary axis. There, the Green’s tensor shows greater numerical stability due to its decaying rather than oscillating character.

The multipole-multipole interaction strength ${\xi }_{\alpha \beta }$ is a generalization of the dipole-dipole coupling which in free space scales as ${R}^{-3}$, $R$ being the interatomic distance. Contrary, a modified photonic environment, e.g., in a photonic crystal, near a nanoparticle or a nanowire, might allow not only for stronger interactions but also for extended interaction distances^9^. Due to the enhancement of off-diagonal elements of the Green’s tensor, long-range coupling of multipoles or, in general, arbitrary nonparallel orientations may be enabled. Due to the strong field localization, corrections beyond the electric dipole approximation in such systems may be significant, and coupling of different multipoles is possible. Interference of different interaction components may lead to further enhancement or suppression of interaction strength, resulting in a corresponding modification of interaction distance. Please note that due to the large width of the peak in the density of states, assumed in Section 2 of the Supplementary Information, atomic systems with slightly different transition frequencies may in general be coupled.

The dissipators in Eq. (14) include emission rates of an individual, ${\alpha }^{th}$ atomic system ${\gamma }_{\alpha \alpha }$ (reducing to $\gamma$ from the previous Section in case of a single system), and collective decay rates ${\gamma }_{\alpha \beta }$. They arise because each atomic system is capable of modifying the photonic environment of the others and they are defined as

19 $${\gamma }_{\alpha \beta }=2\sum _{mj}\left\{{R}_{mj}\left(\bar{\omega }\right)\pi {\bar{\omega }}^{2}{\rm{Im}}\ {G}_{mj}\left({\bf{r}}{\prime} ,{\bf{r}},\bar{\omega }\right){| }_{\begin{array}{c}{\bf{r}}={{\bf{r}}}_{\beta },\\ {\bf{r}}{\prime} ={{\bf{r}}}_{\alpha }\end{array}}-{\mathscr{P}}{\int }_{0}^{\infty }d\omega {I}_{mj}\left(\omega \right)\frac{{\omega }^{2}}{\omega -\bar{\omega }}{\rm{Im}}\ {G}_{mj}\left({\bf{r}}{\prime} ,{\bf{r}},\omega \right){| }_{\begin{array}{c}{\bf{r}}={{\bf{r}}}_{\beta }\end{array},{\bf{r}}{\prime} ={{\bf{r}}}_{\alpha }}\right\},$$

valid also for $\beta =\alpha$, which yields ${I}_{mj}\left(\omega \right)=0$. Again, using Eq. S17 of the Supplementary Information we can simplify the expression to

20 $$\begin{array}{rcl}{\gamma }_{\alpha \beta } & = & 2\pi {\bar{\omega }}^{2}\sum _{mn}\left\{{\left.{R}_{mn}^{\alpha \beta }\left(\bar{\omega }\right){\rm{Im}}{G}_{mn}\left({\bf{r}}{\prime} ,{\bf{r}},\bar{\omega }\right)\right|}_{\begin{array}{c}{\bf{r}}={{\bf{r}}}_{{\boldsymbol{\beta }}},\\ {\bf{r}}{\prime} ={{\bf{r}}}_{\alpha }\end{array}}-{\left.{I}_{mn}^{\alpha \beta }\left(\bar{\omega }\right){\rm{Re}}{G}_{mn}\left({\bf{r}}{\prime} ,{\bf{r}},\bar{\omega }\right)\right|}_{\begin{array}{c}{\bf{r}}={{\bf{r}}}_{{\boldsymbol{\beta }}},\\ {\bf{r}}{\prime} ={{\bf{r}}}_{\alpha }\end{array}}\right\}\\  &  & -2{\int }_{0}^{\infty }d\omega \frac{{\omega }^{2}\bar{\omega }}{{\omega }^{2}+{\bar{\omega }}^{2}}{\rm{Re}}\left[{\left.{I}_{mn}^{\alpha \beta }\left(i\omega \right){G}_{mn}\left({\bf{r}}{\prime} ,{\bf{r}},\omega \right)\right|}_{\begin{array}{c}{\bf{r}}={{\bf{r}}}_{{\boldsymbol{\beta }}},\\ {\bf{r}}{\prime} ={{\bf{r}}}_{\alpha }\end{array}}\right]\end{array}$$

Please note that for identical emitters this problem can be discussed in terms of Dicke superradiance, and would be a straightforward generalization of results of ref. ^47^.

### Examples

In this section we apply the formulas derived above first to the case of a homogeneous background dielectric and second to an exemplary selected nanostructured environment into which the atomic system is placed. In the first considered case, our goal is to retrieve familiar scaling of different multipolar components of spontaneous emission rates with different powers of refractive index^48,49^. In the latter case, we demonstrate that in a suitably engineered environment contributions beyond electric dipole may have a significant impact on the atomic system’s dynamics.

### Homogeneous background medium

In a homogeneous, isotropic, and infinitely extended medium the Green’s tensor takes the form

21 $${\bf{G}}({\bf{R}},\omega )=\left({\bf{1}}+\frac{ikR-1}{{k}^{2}{R}^{2}}{\bf{1}}+\frac{3-3ikR-{k}^{2}{R}^{2}}{{k}^{2}{R}^{4}}{\bf{RR}}\right)\frac{{e}^{ikR}}{4\pi R},$$

where $R=\left|{\bf{R}}\right|=\left|{\bf{r}}-{\bf{r}}{\prime} \right|$ is the distance between the source and observation point where the field is to be evaluated, and the wave number in the homogenous medium reads $k=\frac{\omega }{c}\sqrt{{\boldsymbol{\varepsilon }}(\omega )}=\frac{\omega }{c}n(\omega )$, $n(\omega )$ being a position-independent refractive index.

In Section 4 of the Supplementary Information we show that away from the medium resonances the imaginary part of the Green’s tensor is diagonal in the limit of $R\to 0$ with

22 $$\mathop{{\rm{lim}}\,}\limits_{R\to 0}{\rm{Im}}\ {G}_{jk}({\bf{R}},\omega )=\frac{{k}^{3}}{60\pi }{R}_{j}{R}_{k},$$

for $k\ne j$, and is exactly 0 for $R=0$. The diagonal elements however, are finite and read as

23 $${\rm{Im}}\ {G}_{jj}({\bf{R}},\omega )=\frac{k}{6\pi }-\frac{{k}^{3}}{30\pi }{R}^{2}+\frac{{k}^{3}}{60\pi }{R}_{j}{R}_{j}+O({R}^{4}).$$

Inserting the limit at $R\to 0$ in Eq. (9), we retrieve the Weisskopf-Wigner result for the electric dipole contribution to spontaneous emission

24 $${\gamma }_{{\rm{ED}}}=\frac{n{\omega }_{0}^{3}| d{| }^{2}}{3\pi \hslash {\varepsilon }_{0}{c}^{3}}.$$

Higher-order terms may be evaluated based on derivatives found in Eqs. S27 & S28 of the Supplementary Information.

25 $$\begin{array}{lll}{\gamma }_{{\rm{MD}}} & = & \frac{{n}^{3}{\omega }^{3}{\left|{\bf{m}}\right|}^{2}}{3\pi \hslash {{\boldsymbol{\varepsilon }}}_{0}{c}^{5}},\end{array}$$

26 $$\begin{array}{lll}{\gamma }_{{\rm{EQ}}} & = & \frac{{n}^{3}{\omega }^{5}{\sum }_{mn}{\left|{Q}_{mn}\right|}^{2}}{10\pi \hslash {{\boldsymbol{\varepsilon }}}_{0}{c}^{5}}.\end{array}$$

As the result, we find the familiar result for transition rates *via* higher-order channels^48,51^, which scale with the third power of the refractive index^48,49^. Similarly, multipole-multipole interaction terms can be retrieved.

#### Pair of metallic nanospheres

**Figure 1:**
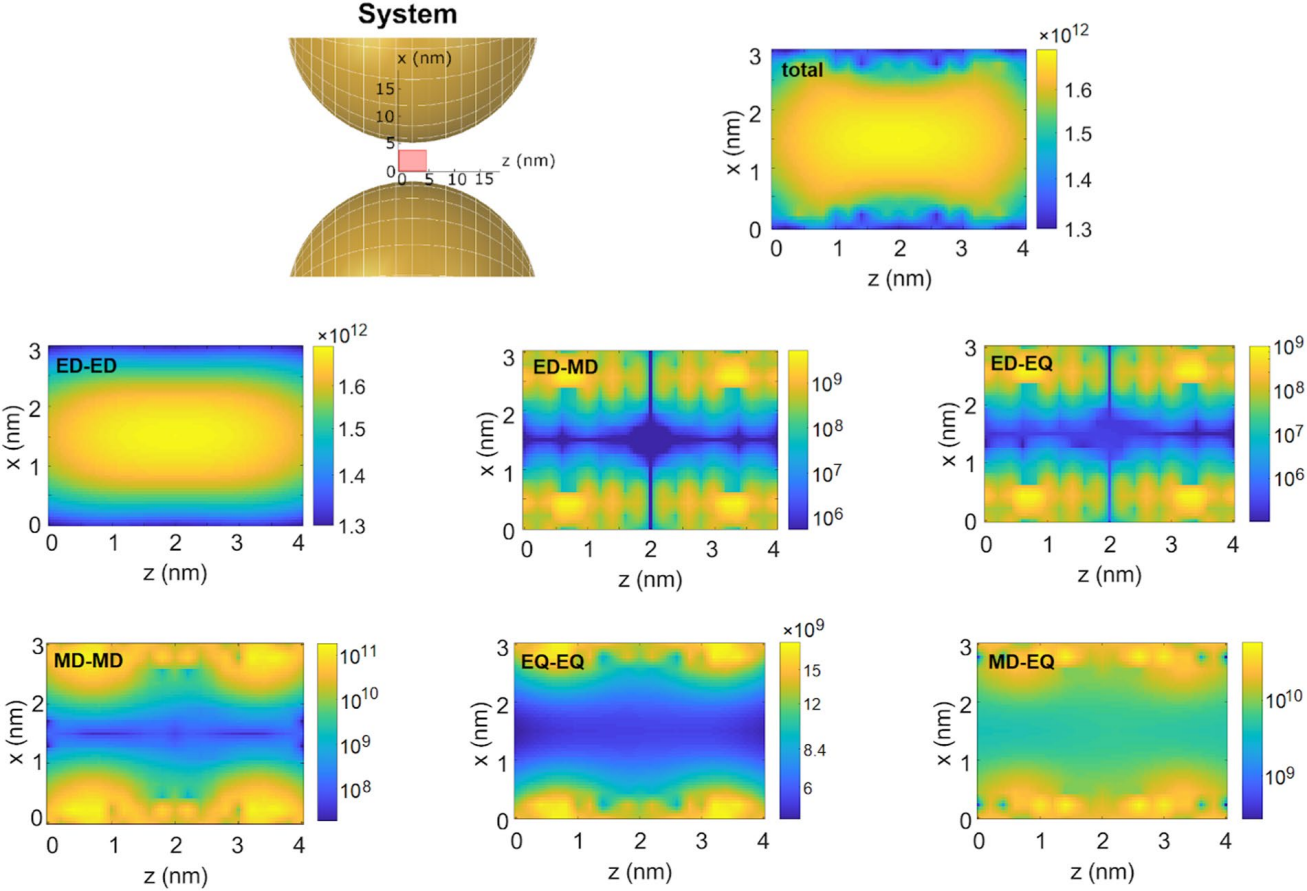
Example nanostructures and spontaneous emission rates. The nanostructures are two silver nanospheres of 40 nm diameter, separated by a 6 nm gap. The pink rectangle indicates the grid on which the Green’s tensor is evaluated. Spontaneous emission of an atomic system is shown as a function of its position within the pink rectangle. The total rates and components in different multipolar channels are given. ED-ED corresponds to the “pure” electric-dipole channel, while ED-MD is the interference between the electric and magnetic dipoles, etc. All rates in Hz. Figure created with use of MNPBEM17 toolbox^50^ for MATLAB (version R2019a). Note that logarythmic scale is used in all panels.

To offer also an example of a structured photonic environment, we consider here a pair of silver nanospheres of 40 nm diameter, separated by a 6 nm gap inside of which an atomic system is positioned. The chosen coordinate frame is shown in the system sketch in Fig. 1. The Green’s tensor was calculated using the MNPBEM toolbox for MATLAB^50^: a Maxwell equation solver based on the Boundary Element Method^52–54^. Full Maxwell equations were solved. Silver was modeled using data from ref. ^55^. The tensor is calculated at the frequency $${\omega }_{0}=2\pi \times 789$$$${{\rm{ps}}}^{-1}$$ on a $$4\ {\rm{nm}}\times 3\ {\rm{nm}}$$ grid located in the symmetry plane $$y=0$$ between the nanospheres, as marked by the rectangle in the system sketch in Fig. 1. The Green’s tensor’s elements and derivatives are presented in the Supplementary Information.

We now consider a two-level atomic system with transition frequency ${\omega }_{0}$. We choose a transition electric dipole moment to be oriented along the $x$ axis, with a length of ${d}_{x}=e{a}_{0}$, where $e$ stands for the elementary charge and ${a}_{0}$ is the Bohr radius. The magnetic transition dipole moment parallel to the $z$ axis is ${m}_{z}=2i{\mu }_{B}$, with ${\mu }_{B}$ standing for Bohr magneton. The electric transition quadrupole moment in the $xy$ plane is set to ${Q}_{xy}={Q}_{yx}=e{a}_{0}^{2}$. The chosen values correspond to all moments equal to 1 atomic unit, i.e. values characteristic for atoms and molecules. The transition rates depend on the position of the atomic system with respect to the nanospheres, and are shown in Fig. 1. We only consider the atomic system’s positions in the rectangle from the system sketch in Fig. 1. In free space, the rates through respective channels are given by Eqs. (24–26)) with $n=1$ and read ${\gamma }_{{\rm{ED,fs}}}\approx 37$ MHz, ${\gamma }_{{\rm{MD,fs}}}\approx 2.0$ kHz and ${\gamma }_{{\rm{EQ,fs}}}\approx 17$ Hz. This means that the ED channel dominates the emission, and if it is present the visibility of the other channels is suppressed. In general, in the gap between the nanospheres the enhanced transition rates in all channels exceed the free-space values by several orders of magnitude: up to 4 in case of the ED and even 7 (8) for the MD (EQ), improving the impact of the higher order channels with respect to the dominant electric dipole. As a result, the channels beyond the electric dipole make a significant contribution to the overall transition rate. This contribution includes the interference channels: for the studied nanostructured geometry and orientations of the multipolar moments we find the interference between the MD and EQ to be relatively strong, while the interference with the ED channel is weaker. However, in general other geometries or orientations of the atomic systems could be considered where these channels, here weak, could be tuned^28^. Among possible applications this suggests potential for enhancement of optical activity, in particular circular dichroism. Among the two considered higher-order channels, the magnetic dipole transition channel dominates by two orders of magnitude over the electric quadrupole one. However, the latter is manifested through interference, which we find always destructive in the investigated region, and whose contribution to the transition rate in channels beyond the ED is of the order of $10 \%$.

**Figure 2:**
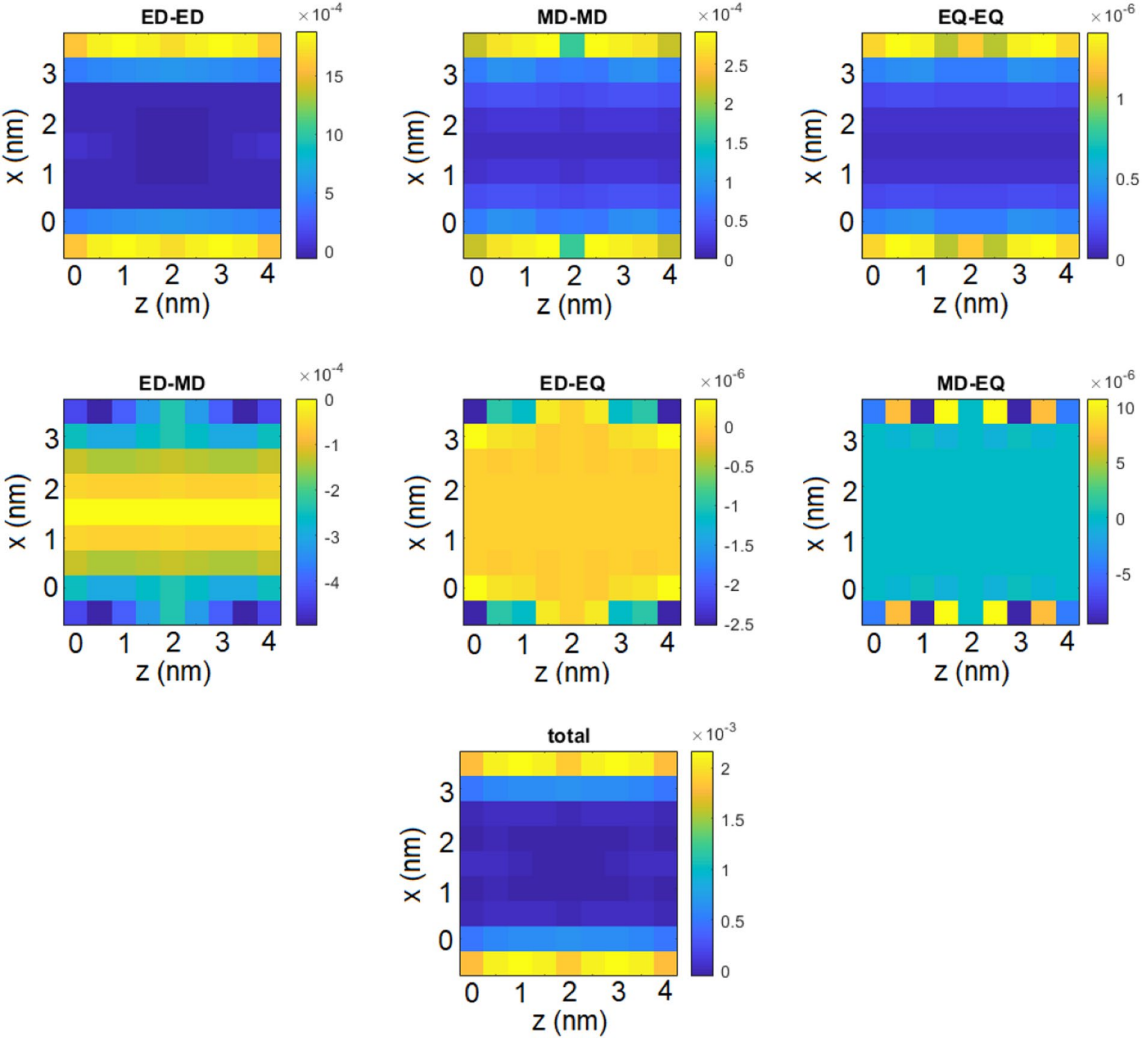
Shift $\delta$ of an atomic system’s transition frequency for selected positions within the pink rectangle in the system sketch in Fig. 1. The first row corresponds to the pure multipolar channels, while the second row displays the interference terms. The total shift is the sum of all components and is shown at the bottom. All shifts in eV. Figure created with use of MNPBEM17 toolbox^50^ for MATLAB (version R2019a).

We now evaluate the correction of the frequency shift, i.e. the Lamb shift, of the atomic system coupled to the quantum vacuum near the two nanospheres. Please note that the full Green’s tensor diverges at the origin. However, we are only interested to evaluate the correction of the shift with respect to free space, determined by the scattered part of the Green’s tensor. The result obtained for selected positions within the gap is shown in Fig. 2. The relative values of the shifts $\delta  < 1{0}^{-3}{\omega }_{0}$ in the investigated domain are dominated by the electric-dipole and the comparable magnetic-dipole channel. Please note the opposite sign of the interference term between the two.

**Figure 3:**
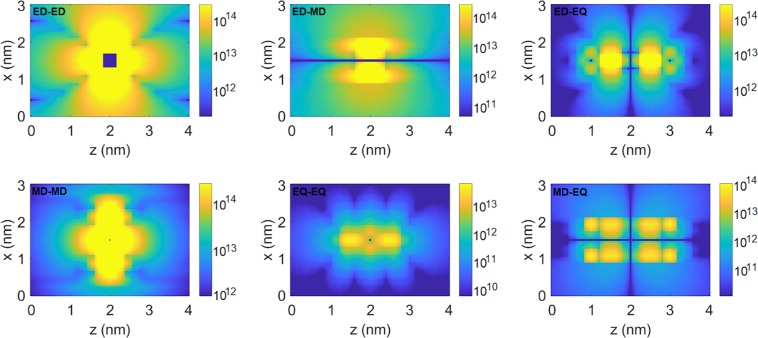
Absolute values of interaction strengths $| \xi |$ between two atomic systems, one of which is positioned at the centre of the simulation domain, in function of position of the other system. All strengths in Hz. Figure created with use of MNPBEM17 toolbox^50^ for MATLAB (version R2019a).

**Figure 4:**
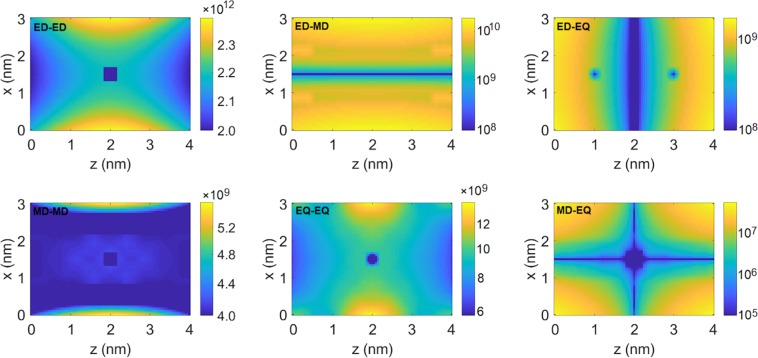
Absolute values of collective decay rates ${\gamma }_{12}$ of two atomic systems, one of which is positioned at the centre of the simulation domain, in function of position of the other system. All rates in Hz. Figure created with use of MNPBEM17 toolbox^50^ for MATLAB (version R2019a).

Finally, we evaluate the interaction strengths $| \xi |$ (Fig. 3) and collective emission rates ${\gamma }_{12}$ (Fig. 4) for a pair of atomic systems, one of which is positioned at the center of the domain in between the nanospheres, while the position of the other is swept across the simulation domain. The evaluated interaction strengths reach tens of THz throughout the simulation domain. Please note that atomic systems separated by very small distances might interact through mechanisms other than the optical one, e.g. through electron exchange. The total interaction strength and collective decay rate is a sum of contributions from all channels. Please note that in the case of interaction strength we find the contributions from all investigated multipoles to be significant. Contrary, the collective decay rates are dominated by the ED channel.

## Discussion

We have studied dynamics of atomic systems coupled to a photonic environment in its vacuum state. The environment is described in terms of the electromagnetic Green’s tensor and the interaction contributions beyond the paradigmatic electric dipole approximation have been included. The derived formalism allows us to evaluate dynamical parameters characterizing optical properties of atomic systems: both the individual and the collective contributions to energy shifts and decay rates. Inclusion of terms beyond the electric dipole approximation allows us to study the role of higher multipolar channels, including enhancement or suppression through interference of different interaction mechanisms. Examples of phenomena to be investigated are optical activity, multipole-multipole interactions between atomic systems and spontaneous emission suppression due to interference of different mutipolar channels in tailored photonic environments.

## Methods

Our approach is based on quantization scheme introduced in refs. ^39,42^. To derive equations presented in this work we apply the Markovian approximation, as described in detail in the Supplementary Information available online.

Green’s tensor corresponding to the pair of metallic nanospheres in the Examples section was calculated using the MNPBEM toolbox for MATLAB^50^: a Maxwell equation solver based on the Boundary Element Method^52–54^. Full Maxwell equations were solved. Silver was modeled using data from ref. ^55^.

## Supplementary information

Supplementary Information.
