# Supplementary material for: Interaction of atomic systems with quantum vacuum beyond electric dipole approximation
Source: Sci Rep. 2020 Apr 3;10:5879. doi: 10.1038/s41598-020-62629-0 (PMC7125098; doi:10.1038/s41598-020-62629-0)
Supplement: Supplementary file 1 — Supplementary Information. [file 41598_2020_62629_MOESM1_ESM.pdf]

Supplementary Information for "Interaction of atomic systems with quantum vacuum beyond electric dipole approximation"

Miriam Kosik[1,*], Oleksandr Burlayenko[2], Carsten Rockstuhl[3], Ivan Fernandez-Corbaton[3], and Karolina Słowik[1,*]

[1]Institute of Physics, Faculty of Physics, Astronomy and Informatics, Nicolaus Copernicus University in Toruń, Grudziadzka 5, 87-100 Torun, Poland
[2]Department of Physics and Technology, V.N. Karazin Kharkiv National University, Kharkiv, Ukraine
[3]Institute of Theoretical Solid State Physics, Karlsruhe Institute of Technology, 76131 Karlsruhe, Germany and Institute of Nanotechnology, Karlsruhe Institute of Technology, 76021 Karlsruhe, Germany
[*]mkosik@doktorant.umk.pl, karolina@fizyka.umk.pl

ABSTRACT

The following supplementary material is provided in different sections:
1. Derivation of the effective atomic dynamics for single atomic system
2. Generalization to the case of multiple atomic systems
3. Replacing principal value integrals
4. Evaluation of Green's tensor at source's location in homogeneous media
5. Green's tensor of a pair of metallic nanoparticles

1 Derivation of the effective atomic dynamics for single atomic system

We begin with the Heisenberg equations for both atomic systems and fields $i\hbar\dot{O} = [O, \mathscr{H}]$ where O is an arbitrary operator and $\mathscr{H} = \mathscr{H}_0 + \mathscr{H}_{\text{field}} + \mathscr{H}_{\text{int}}$ is the total Hamiltonian. The equations read

$$\dot{\sigma} = -i\omega_0\sigma - \frac{i}{\hbar}\sigma_z\left(\mathbf{d}^\dagger\cdot\mathbf{E}_0^{(+)} + \mathbf{m}^\dagger\cdot\mathbf{B}_0^{(+)} + \mathbf{Q}^\dagger : \nabla\mathbf{E}_0^{(+)}\right) \tag{S1}$$

$$\dot{\sigma}_z = \frac{2i}{\hbar}\left\{\sigma^\dagger\left(\mathbf{d}^\dagger\cdot\mathbf{E}_0^{(+)} + \mathbf{m}^\dagger\cdot\mathbf{B}_0^{(+)} + \mathbf{Q}^\dagger : \nabla\mathbf{E}_0^{(+)}\right) - \left(\mathbf{E}_0^{(-)}\cdot\mathbf{d} + \mathbf{B}_0^{(-)}\cdot\mathbf{m} + \nabla\mathbf{E}_0^{(-)} : \mathbf{Q}\right)\sigma\right\} \tag{S2}$$

$$\begin{aligned}\dot{f}_{\omega,j} &= -i\omega f_{\omega,j} + \frac{i}{\hbar}\left[f_{\omega,j}, \mathbf{E}_0^{(-)}\cdot\mathbf{d} + \mathbf{B}_0^{(-)}\cdot\mathbf{m} + \nabla\mathbf{E}_0^{(-)} : \mathbf{Q}\right]\sigma \\ &= -i\omega f_{\omega,j}(\mathbf{r}) + \sqrt{\frac{\operatorname{Im}\varepsilon(\mathbf{r},\omega)}{\hbar\pi\varepsilon_0}}\frac{\omega^2}{c^2}\sum_m D_m^{r''}(\omega)G_{mj}^\star(\mathbf{r}'',\mathbf{r},\omega)\,|_{\mathbf{r}''=\mathbf{r}_0}\sigma,\end{aligned} \tag{S3}$$

where we have used the commutation relations of atomic Pauli operators and of bosonic field operators given by Eqs. (4) of the main text. We have used the representation of double dot product $\nabla\mathbf{E}^{(-)}(\mathbf{r}) : \mathbf{Q} = \sum_{ij} Q_{ji}\partial_i E_j^{(-)}(\mathbf{r})$ and of rotation $\left[\nabla\times\mathbf{E}^{(-)}(\mathbf{r})\right]_i = \sum_{jk}\varepsilon_{ijk}\partial_{r_j}E_k^{(-)}(\mathbf{r})$. We now formally integrate Eq. (S3) to obtain

$$f_{\omega,j}(t) = f_{\omega,j}^{\text{free}}(\mathbf{r}) + \sqrt{\frac{\operatorname{Im}\varepsilon(\mathbf{r},\omega)}{\hbar\pi\varepsilon_0}}\frac{\omega^2}{c^2}\left\{\sum_m D_m^{r''}(\omega)G_{mj}^\star(\mathbf{r}'',\mathbf{r},\omega)\,|_{\mathbf{r}''=\mathbf{r}_0}\right\}\int_0^t dt'\sigma(t')e^{-i\omega(t-t')} \tag{S4}$$

where $f_{\omega,j}^{\text{free}}(t)$ describes the free field, and the integral accounts for the influence of the atomic system on the field. Since we focus on the atomic dynamics, the above result is inserted in the place of fields in Eqs. (S1-S2). The resulting integro-differential equations can be simplified if the coupling with the environment is weak: In the free-atomic-system case, the operator $\sigma(t) = \sigma(0)e^{-i\omega_0 t}$ evolves freely. The photonic environment, which represents vacuum fluctuations, is characterized by a set of modes continuously distributed in frequencies and weakly coupled to the atomic system. Such environment introduces small modifications to the atomic evolution, which can be quantified in terms of a slowly varying envelope $\tilde{\sigma}(t)$ modulated upon the

free oscillations: $\sigma(t) = \tilde{\sigma}(t)e^{-i\omega_0 t}$. The assumption in the Markovian approximation is that the envelope changes little over the time interval ω_0^{-1} around $t' \approx t$, where the oscillating term takes significant values. This yields

$$\begin{aligned}
&\int_0^t dt' \sigma\left(t'\right) e^{-i\omega(t-t')} = e^{-i\omega_0 t} \int_0^t dt' \tilde{\sigma}\left(t'\right) e^{-i(\omega-\omega_0)(t-t')} \\
&\approx \sigma(t) \int_0^\infty e^{i(\omega-\omega_0)\tau} d\tau \\
&= \sigma(t) \left\{\pi\delta(\omega-\omega_0) + i\mathscr{P}\left[(\omega-\omega_0)^{-1}\right]\right\}.
\end{aligned} \tag{S5}$$

Due to this time-scale mismatch, the time of interest $t \gg \omega_0^{-1}$ and we could replace the upper limit of the above integral with infinity. In the last step we have used the Sokhotski–Plemelj theorem[1]. The symbol $\mathscr{P}$ denotes the Cauchy principal value. The interpretation is that the evolution of the system is affected only by its present state, and memory effects are negligible.

Please note that here and in the following part of the derivation it is crucial to consequently keep normal operator ordering, as it naturally follows from the Hamiltonian in Eq. (6) of the main text. This is because the Markovian approximation in general affects the commutation relations, and atomic and field operators are no longer independent.

Having inserted Eq. (S4) into Eqs. (S1) and (S2) and applying the Markovian approximation given by Eq. (S5), we arrive into Eqs. (7) and (8) of the main text. We have additionally used the Green's tensor property[2]

$$\frac{\omega^2}{c^2} \int d^3r \operatorname{Im}\varepsilon(\mathbf{r},\omega) \sum_n G_{\mathrm{mn}}(\mathbf{r}_1,\mathbf{r},\omega) G^\star_{\mathrm{pn}}(\mathbf{r}_2,\mathbf{r},\omega) = \operatorname{Im} G_{\mathrm{mp}}(\mathbf{r}_1,\mathbf{r}_2,\omega), \tag{S6}$$

and the reciprocity relation

$$G_{ij}\left(\mathbf{r}',\mathbf{r},\omega\right) = G_{ji}\left(\mathbf{r},\mathbf{r}',\omega\right). \tag{S7}$$

2 Generalization to the case of multiple atomic systems

We begin with the Hamiltonian given by Eq. (13) of the main text and repeat steps corresponding to Eqs. (S1-S4). To perform the Markovian approximation, we note that we allow few atomic species to oscillate with in general different frequencies ω_α. We assume that these frequencies are relatively close to each other $\Delta_\omega \equiv \max_{\alpha,\alpha'} |\omega_\alpha - \omega'_\alpha| \ll \bar{\omega}$, where $\bar{\omega}$ is the average transition frequency. In this case, in the joint response of the atomic systems we expect beating: fast oscillations with average frequency $\bar{\omega}$ are modulated with an envelope varying at timescales of the order of Δ_ω^{-1}. Additionally, we assume that the Green's tensor has a relatively broad peak at the frequency range of interest, covering all atomic frequencies, and assume that nevertheless the two-level approximation for each system still holds. We choose a corresponding ansatz to represent atomic operators for each α: $\sigma_\alpha(t) = \tilde{\sigma}_\alpha(t)e^{-i\bar{\omega}t}$, i.e. we separate the fast oscillation and include the slowly varying envelope in $\tilde{\sigma}_\alpha(t)$. With this ansatz we can rewrite Eq. (S5) in the multi-atomic-system case. As a result, using also Eqs. (S6,S7), we arrive at Eqs. (14 & 15) of the main text. Effective coupling strengths $\xi_{\alpha,\beta}$ and collective decay rates $\gamma_{\alpha.\beta}$ are derived as the result, expressed through the following operators

$$\begin{aligned}
R^{\alpha\beta}_{mn}(\omega) &= \frac{1}{\hbar\pi\varepsilon_0 c^2}\Bigg\{\operatorname{Re}\left[d^\star_{\alpha,m} d_{\beta,n}\right] + \operatorname{Re}\left[d^\star_{\alpha,m}\sum_l\left(Q_{\beta,nl} + i\omega^{-1}\sum_s \varepsilon_{sln} m_{\beta,s}\right)\right]\partial_{r_l} + \operatorname{Re}\left[\sum_k\left(Q^\star_{\alpha,mk} - i\omega^{-1}\sum_p \varepsilon_{pkm} m^\star_{\alpha,p}\right) d_{\beta,n}\right]\partial_{r'_k} \\
&\quad + \operatorname{Re}\left[\sum_k\left(Q^\star_{\alpha,mk} - i\omega^{-1}\sum_p \varepsilon_{pkm} m^\star_{\alpha,p}\right)\right]\left[\sum_l\left(Q_{\beta,nl} + i\omega^{-1}\sum_s \varepsilon_{sln} m_{\beta,s}\right)\right]\partial_{r'_k}\partial_{r_l}\Bigg\} \\
&\equiv \frac{1}{\hbar\pi\varepsilon_0 c^2}\operatorname{Re}\left[D^{r'}_{\alpha,m}{}^\dagger(\omega) D^r_{\beta,n}(\omega)\right]
\end{aligned} \tag{S8}$$

$$\begin{aligned}
I^{\alpha\beta}_{mn}(\omega) &= \frac{1}{\hbar\pi\varepsilon_0 c^2}\Bigg\{\operatorname{Im}\left[d^\star_{\alpha,m} d_{\beta,n}\right] + \operatorname{Im}\left[d^\star_{\alpha,m}\sum_l\left(Q_{\beta,nl} + i\omega^{-1}\sum_s \varepsilon_{sln} m_{\beta,s}\right)\right]\partial_{r_l} + \operatorname{Im}\left[\sum_k\left(Q^\star_{\alpha,mk} - i\omega^{-1}\sum_p \varepsilon_{pkm} m^\star_{\alpha,p}\right) d_{\beta,n}\right]\partial_{r'_k} \\
&\quad + \operatorname{Im}\left[\sum_k\left(Q^\star_{\alpha,mk} - i\omega^{-1}\sum_p \varepsilon_{pkm} m^\star_{\alpha,p}\right)\right]\left[\sum_l\left(Q_{\beta,nl} + i\omega^{-1}\sum_s \varepsilon_{sln} m_{\beta,s}\right)\right]\partial_{r'_k}\partial_{r_l}\Bigg\} \\
&\equiv \frac{1}{\hbar\pi\varepsilon_0 c^2}\operatorname{Im}\left[D^{r'}_{\alpha,m}{}^\dagger(\omega) D^r_{\beta,n}(\omega)\right]
\end{aligned} \tag{S9}$$

where the operator $D^r_{\alpha,m}(\omega)$ now depends on multipolar moments of the αs emitter.

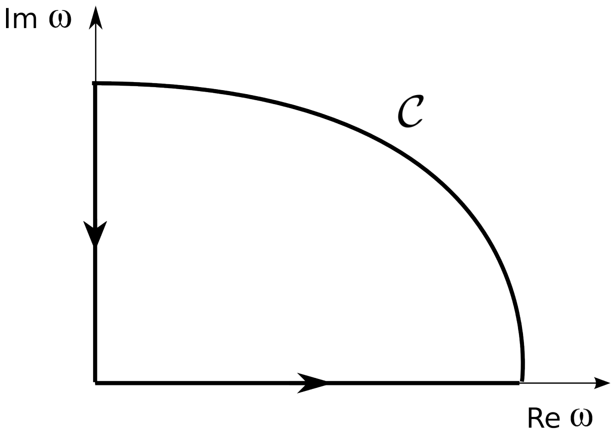

Figure S1. Contour for complex-plane integration.

3 Replacing principal value integrals

For purposes of this section, we note that the real and imaginary components of the product of generalized multipole moments, given by Eqs. (S8 & S9) of Section 2, have the functional dependence on frequency of the form $R_{mn}^{\alpha\beta}(\omega), I_{mn}^{\alpha\beta}(\omega) = f_0 + \frac{1}{\omega} f_1 + \frac{1}{\omega^2} f_2$, where $f_{0,1,2}$ are frequency-independent, real-valued parameters possibly multiplied by spatial differentiation operators to act on Green's tensors. As mentioned before, we assume that the derivatives exist at positions of atomic systems, i.e. these system should not be placed exactly at interfaces of different media. Then, differentiation over spatial coordinates and integration over frequency are interchangeable. With this assumption, the dependence on spatial coordinates plays no further role throughout this section, therefore we use the simplified notation $G_{mn}(\mathbf{r}, \mathbf{r}', \omega) \equiv G_{mn}(\omega)$. We follow and generalize considerations in Ref.[3] to eliminate principle-value integrals from terms describing energy shifts.
Equations (17 & 20) of the main text are expressed through principal-value integrals of the form

$$\begin{aligned} I &= \mathscr{P}\int_0^\infty d\omega \frac{\omega^2}{\omega-\omega_0}\left(f_0 + \frac{1}{\omega}f_1 + \frac{1}{\omega^2}f_2\right)\mathrm{Im}\,G_{mn}(\omega) \\ &= \underbrace{\mathscr{P}\int_0^\infty d\omega \frac{\omega^3}{\omega^2-\omega_0^2}\left(f_0 + \frac{1}{\omega}f_1 + \frac{1}{\omega^2}f_2\right)\mathrm{Im}\,G_{mn}(\omega)}_{I_1} \\ &+ \underbrace{\mathscr{P}\int_0^\infty d\omega \frac{\omega^2}{\omega^2-\omega_0^2}\omega_0\left(f_0 + \frac{1}{\omega}f_1 + \frac{1}{\omega^2}f_2\right)\mathrm{Im}\,G_{mn}(\omega)}_{I_2} \end{aligned} \tag{S10}$$

From Kramers-Kronig relations we have

$$\begin{aligned} \omega_0^2 \mathrm{Re}\,G_{mn}(\omega_0) &= \frac{\omega_0^2}{\pi}\mathscr{P}\int_{-\infty}^\infty d\omega \frac{1}{\omega-\omega_0}\mathrm{Im}\,G_{mn}(\omega) \\ &= \frac{2\omega_0^2}{\pi}\mathscr{P}\int_0^\infty d\omega \frac{\omega}{\omega^2-\omega_0^2}\mathrm{Im}\,G_{mn}(\omega) && \text{(S11)} \\ &= \frac{2}{\pi}\mathscr{P}\int_0^\infty d\omega \frac{\omega^3}{\omega^2-\omega_0^2}\mathrm{Im}\,G_{mn}(\omega), && \text{(S12)} \end{aligned}$$

where relation (S11) follows from the assumption of real-valued Green's propagator in time domain $\mathbf{G}(-\omega^\star) = \mathbf{G}^\star(\omega)$, while Eq. (S12) is justified for large frequencies, for which the peak around ω_0 is shifted sufficiently far away from 0[3]. The resulting expression allows us to simplify the first term in Eq. (S10) to the form

$$I_1 = \frac{\pi}{2}\omega_0^2\left(f_0 + \frac{1}{\omega}f_1 + \frac{1}{\omega^2}f_2\right)G'_{mn}(\omega_0). \tag{S13}$$

The second term in Eq. (S10) can be rewritten as

$$I_2 = \mathrm{Im}\left\{ \mathscr{P}\int_0^\infty d\omega \frac{\omega^2}{\omega^2-\omega_0^2}\omega_0 \left[f_0 + \frac{1}{\omega}f_1 + \frac{1}{\omega^2}f_2\right] G_{mn}(\omega) \right\}. \tag{S14}$$

The principal value above can be resolved as

$$\mathscr{P}\frac{\omega_0}{\omega^2-\omega_0^2} = \frac{1}{2}\lim_{\varepsilon\to 0}\left(\frac{1}{\omega-\omega_0-i\varepsilon} - i\pi\delta(\omega-\omega_0) - \frac{1}{\omega+\omega_0-i\varepsilon} + i\pi\delta(\omega+\omega_0)\right), \tag{S15}$$

which yields

$$\begin{aligned} I_2 &= \mathrm{Im}\left\{\int_0^\infty d\omega \frac{1}{2}\lim_{\varepsilon\to 0}\left(\frac{1}{\omega-\omega_0-i\varepsilon} - \frac{1}{\omega+\omega_0-i\varepsilon}\right)\omega^2\left[f_0+\frac{1}{\omega}f_1+\frac{1}{\omega^2}f_2\right]G_{mn}(\omega)\right\} \\ &\quad -\mathrm{Re}\left\{\frac{\pi}{2}\omega_0^2\left[f_0+\frac{1}{\omega_0}f_1+\frac{1}{\omega_0^2}f_2\right]G_{mn}(\omega_0)\right\}. \end{aligned} \tag{S16}$$

Following Ref.[3], we now consider an integral along a closed contour in Fig. S1 . We express the integral along the real semiaxis as a difference of the closed contour integral, integration along the imaginary semiaxis and integral along the curved contour $\mathscr{C}$ sufficiently distant for the integrated function to vanish: $\int_0^\infty \to \oint - \int_{i\infty}^0 - \int_{\mathscr{C}}$. For the closed contour integral we make use of the residue theorem

$$\mathrm{Im}\left\{\oint d\omega \frac{1}{2}\lim_{\varepsilon\to 0}\left(\frac{1}{\omega-\omega_0-i\varepsilon} - \frac{1}{\omega+\omega_0-i\varepsilon}\right)\omega^2\left[f_0+\frac{1}{\omega}f_1+\frac{1}{\omega^2}f_2\right]G_{mn}(\omega)\right\} = \pi\omega_0^2\left[f_0+\frac{1}{\omega_0}f_1+\frac{1}{\omega_0^2}f_2\right]G'_{mn}(\omega_0).$$

Including integration along the imaginary axis and the contribution from I_1, we obtain

$$I = \pi\omega_0^2\left[f_0+\frac{1}{\omega_0}f_1+\frac{1}{\omega_0^2}f_2\right]\mathrm{Re}G_{mn}(\omega_0) + \int_0^\infty d\kappa\kappa^2\left(\frac{\omega_0}{\kappa^2+\omega_0^2}\right)\mathrm{Re}\left\{\left(f_0+\frac{1}{i\kappa}f_1-\frac{1}{\kappa^2}f_2\right)G_{mn}(i\kappa)\right\}. \tag{S17}$$

Finally, we have replaced the principal value integral with an integration along the imaginary axis, where the integrated function in better behaved and more stable numerically.

4 Evaluation of Green's tensor at source's location in homogeneous media

We are interested to find the limit for $R\to 0$ of Eq. (21) of the main text, describing the homogeneous-medium Green's tensor. An off-diagonal element of the tensor is proportional to

$$G_{jk}(R,\omega) \sim \left(3-3ikR-k^2R^2\right)e^{ikR}. \tag{S18}$$

We focus on the case of atomic system's transition far-detuned from medium resonances, in which the refractive index is approximately real. Taylor-expanding the exponent around $R=0$ we find the imaginary part of the element above

$$\begin{aligned} \mathrm{Im}\,G_{jk}(R,\omega) &= \left[\left(3-k^2R^2\right)\left(kR-\frac{k^3R^3}{3!}+\frac{k^5R^5}{5!}-\ldots\right) - 3kR\left(1-\frac{k^2R^2}{2!}+\frac{k^4R^4}{4!}-\ldots\right)\right]\frac{R_jR_k}{4\pi k^2R^5} \\ &= \frac{k^3}{60\pi}R_jR_k + O(R^4). \end{aligned} \tag{S19}$$

Please note that all the lower-order terms vanish identically, i.e. the terms in the square bracket proportional to first and third powers of R. A similar calculation for diagonal terms leads to

$$\mathrm{Im}\,G_{jj}(R,\omega) = \frac{k}{6\pi} - \frac{k^3}{30\pi}R^2 + \frac{k^3}{60\pi}R_jR_j + O(R^4). \tag{S20}$$

We now need to calculate Green's tensor's derivatives in Cartesian coordinates

$$\partial_m \mathrm{Im}\, G_{jk}(R\to 0,\omega) = \frac{k^3}{60\pi}\left(R_k\delta_{mj}+R_j\delta_{mk}\right), \tag{S21}$$

$$\begin{aligned}\partial_m \mathrm{Im}\, G_{jj}(R\to 0,\omega) &= \partial_m\left(-\frac{k^3}{30\pi}\sum_k R_k^2+\frac{k^3}{60\pi}R_j^2\right)\\ &= -\frac{k^3}{15\pi}R_m+\frac{k^3}{30\pi}R_j\delta_{mj}\end{aligned} \tag{S22}$$

Please note that the derivatives of diagonal and off-diagonal elements are comparable. Second-order derivatives are

$$\partial_n\partial_m \mathrm{Im}\, G_{jk}(R\to 0,\omega) = \frac{k^3}{60\pi}\left(\delta_{nk}\delta_{mj}+\delta_{nj}\delta_{mk}\right), \tag{S23}$$

$$\partial_n\partial_m \mathrm{Im}\, G_{jj}(R\to 0,\omega) = -\frac{k^3}{15\pi}\delta_{mn}+\frac{k^3}{30\pi}\delta_{mj}\delta_{nj}. \tag{S24}$$

We now want to make a shift to derivatives over $\mathbf{r}$ and $\mathbf{r}'$. We have $\frac{\partial f}{\partial r_k}=\sum_j \frac{\partial f}{\partial R_j}\frac{\partial R_j}{\partial r_k}$, and similarly for $\mathbf{r}'$ derivatives. In Cartesian coordinates $R_j = r_j - r'_j$, and $R=\sqrt{\sum_j R_j^2}$. Therefore

$$\begin{aligned}\frac{\partial}{\partial r_p}\mathrm{Im}\, G_{jk}(R\to 0,\omega) &= \sum_m \frac{\partial}{\partial R_m}\mathrm{Im}\, G_{jk}(R\to 0,\omega)\delta_{mp}\\ &= \frac{k^3}{60\pi}\left(R_k\delta_{pj}+R_j\delta_{pk}\right),\end{aligned} \tag{S25}$$

and

$$\begin{aligned}\frac{\partial}{\partial r_p}\mathrm{Im}\, G_{jj}(R\to 0,\omega) &= \sum_m \frac{\partial}{\partial R_m}\mathrm{Im}\, G_{jj}(R\to 0,\omega)\delta_{mp}\\ &= -\frac{k^3}{15\pi}R_p+\frac{k^3}{30\pi}R_j\delta_{pj}.\end{aligned} \tag{S26}$$

Computation of second derivatives over primed variables leads to

$$\frac{\partial}{\partial r'_q}\frac{\partial}{\partial r_p}\mathrm{Im}\, G_{jk}(R\to 0,\omega) = -\left[\frac{k^3}{60\pi}\left(\delta_{kq}\delta_{pj}+\delta_{jq}\delta_{pk}\right)\right], \tag{S27}$$

and

$$\frac{\partial}{\partial r'_q}\frac{\partial}{\partial r_p}\mathrm{Im}\, G_{jj}(R\to 0,\omega) = \delta_{pq}\left(\frac{k^3}{15\pi}-\frac{k^3}{30\pi}\delta_{jq}\right). \tag{S28}$$

5 Green's tensor of a pair of metallic nanospheres

The following plots depict the imaginary part of the Green's tensor evaluated on the rectangular grid that is marked in Fig. 1a of the main text. The Green's tensor in each point of the grid is calculated from a source located in the same point. Thus the real part would always be infinite and we only show the imaginary part.

References

1. Blanchard, P. & Brüning, E. *Mathematical Methods in Physics: Distributions, Hilbert Space Operators, and Variational Methods*. Progress in Mathematical Physics (Birkhäuser, 2003).
2. Vogel, W. & Welsch, D.-G. *Quantum Optics* (Wiley-VCH, 2006).
3. Dzsotjan, D., Kästel, J. & Fleischhauer, M. Dipole-dipole shift of quantum emitters coupled to surface plasmons of a nanowire. *Phys. Rev. B* **84**, 075419 (2011).

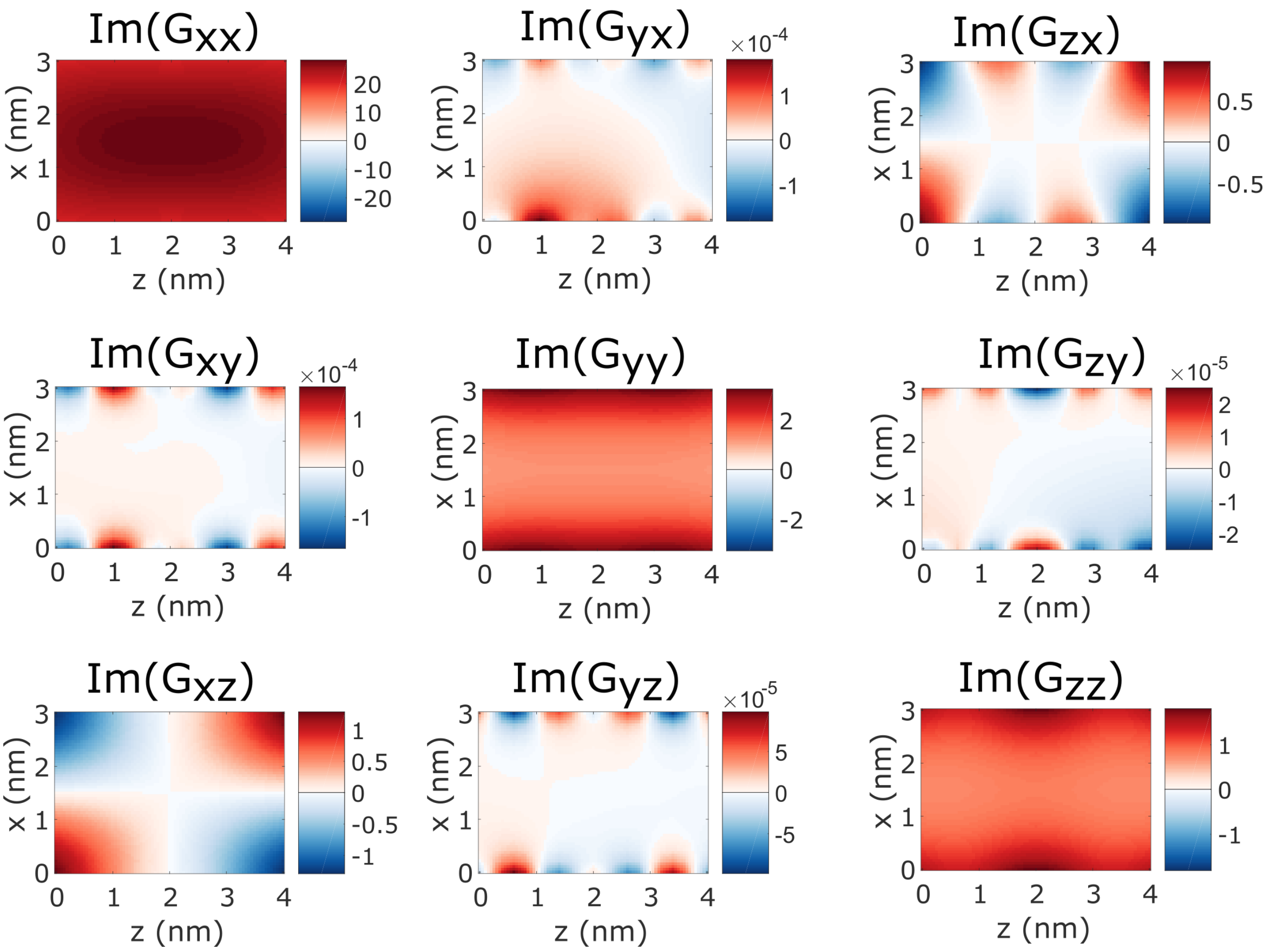

Figure S2. Green's tensor evaluated at the position of source (in nm^{-1}). Figure created in MATLAB R2019a (www.mathworks.com).

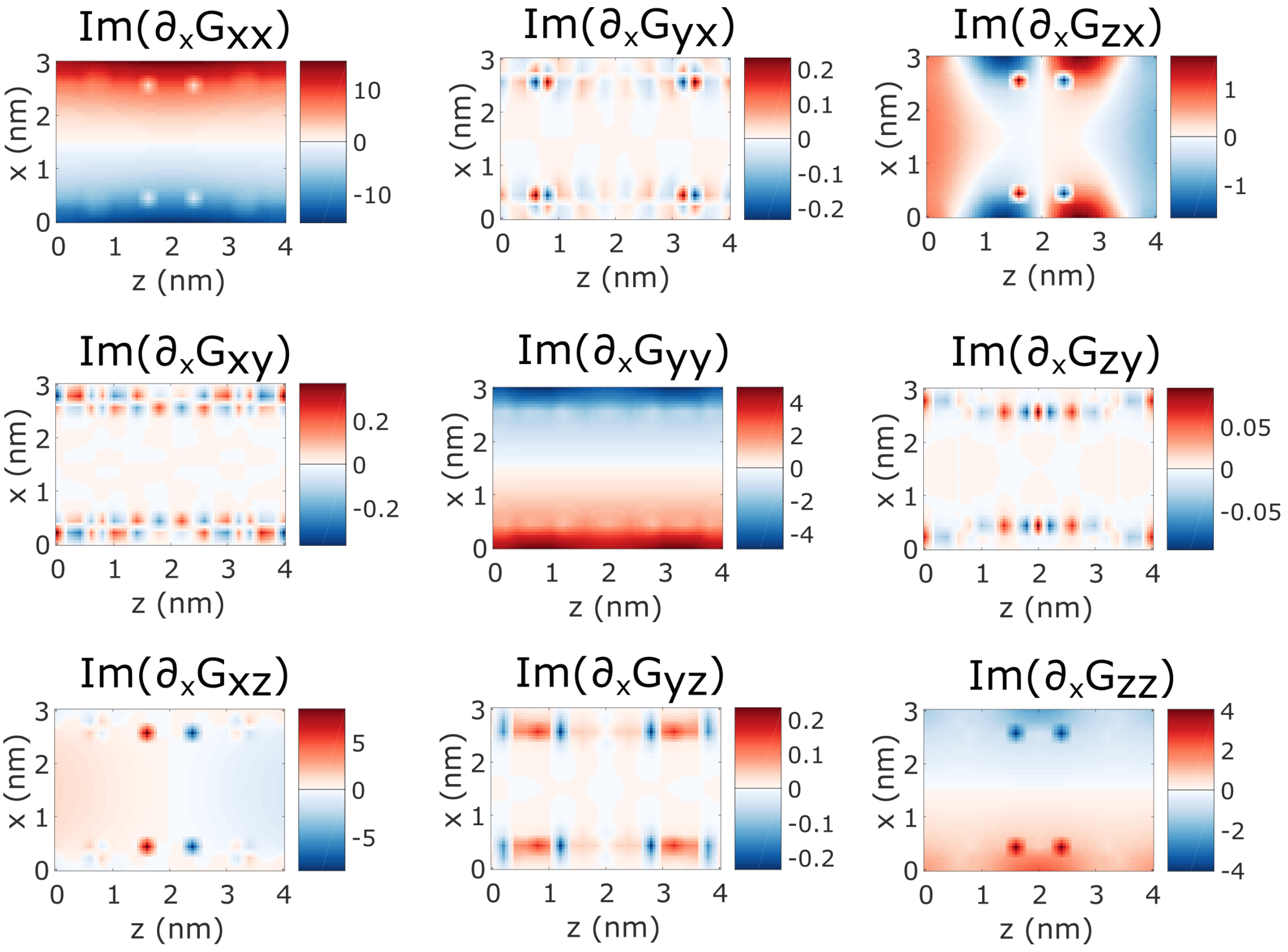

Figure S3. Green's tensor derivative along x direction evaluated at the position of source (in nm^{-2}). Figure created in MATLAB R2019a (www.mathworks.com).

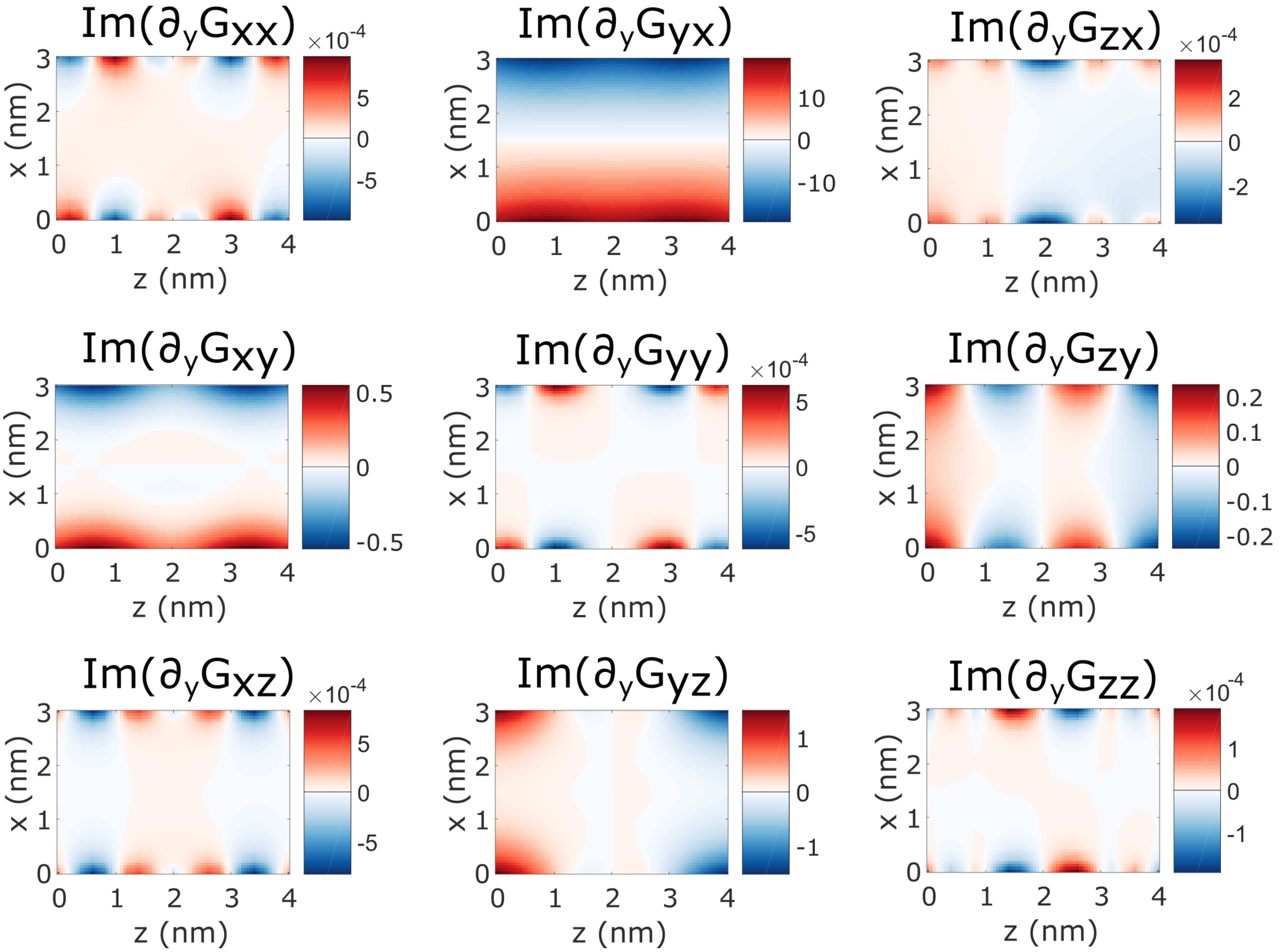

Figure S4. Green's tensor derivative along y direction evaluated at the position of source (in nm^{-2}). Figure created in MATLAB R2019a (www.mathworks.com).

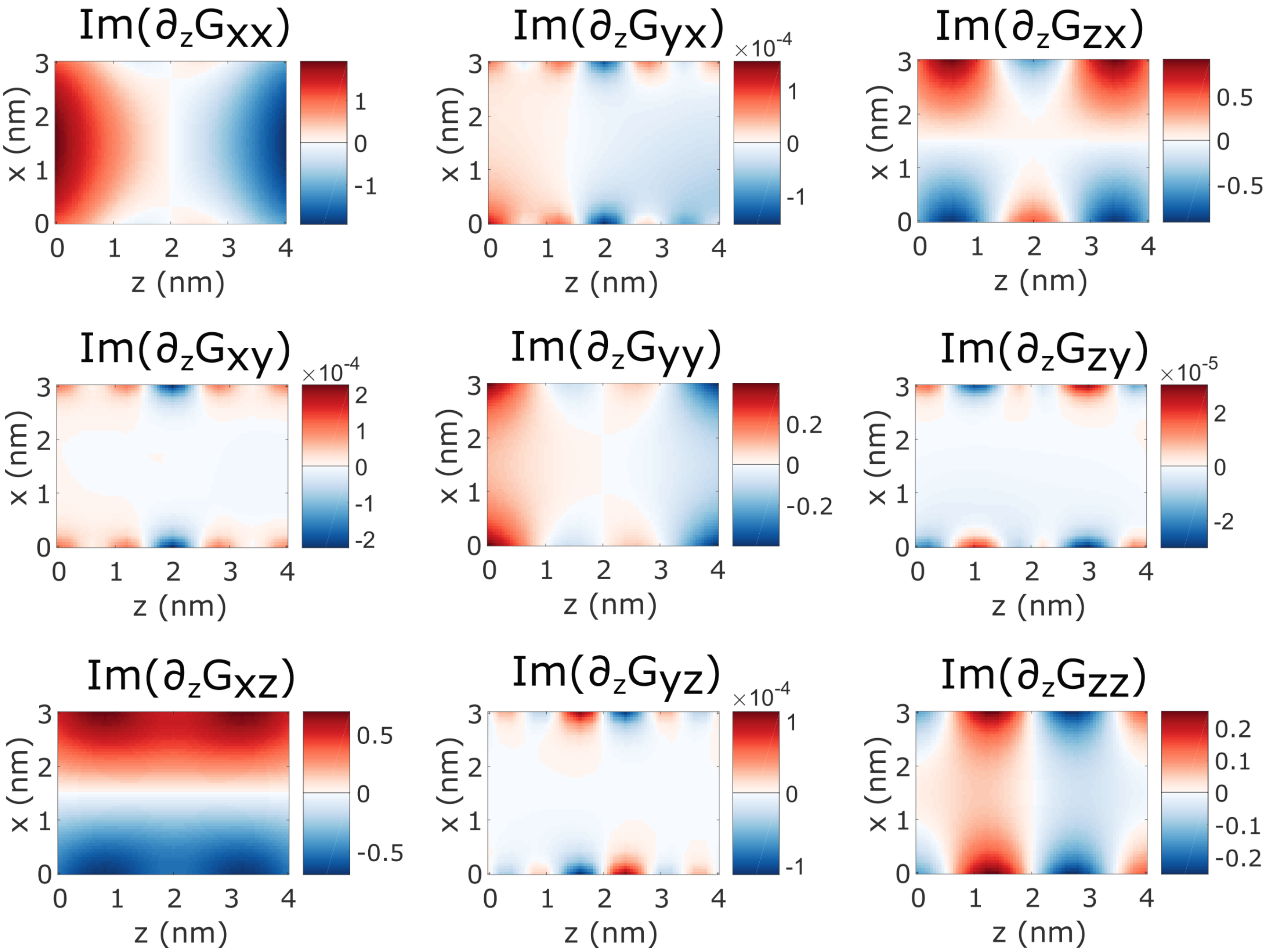

Figure S5. Green's tensor derivative along z direction evaluated at the position of source (in nm^{-2}). Figure created in MATLAB R2019a (www.mathworks.com).

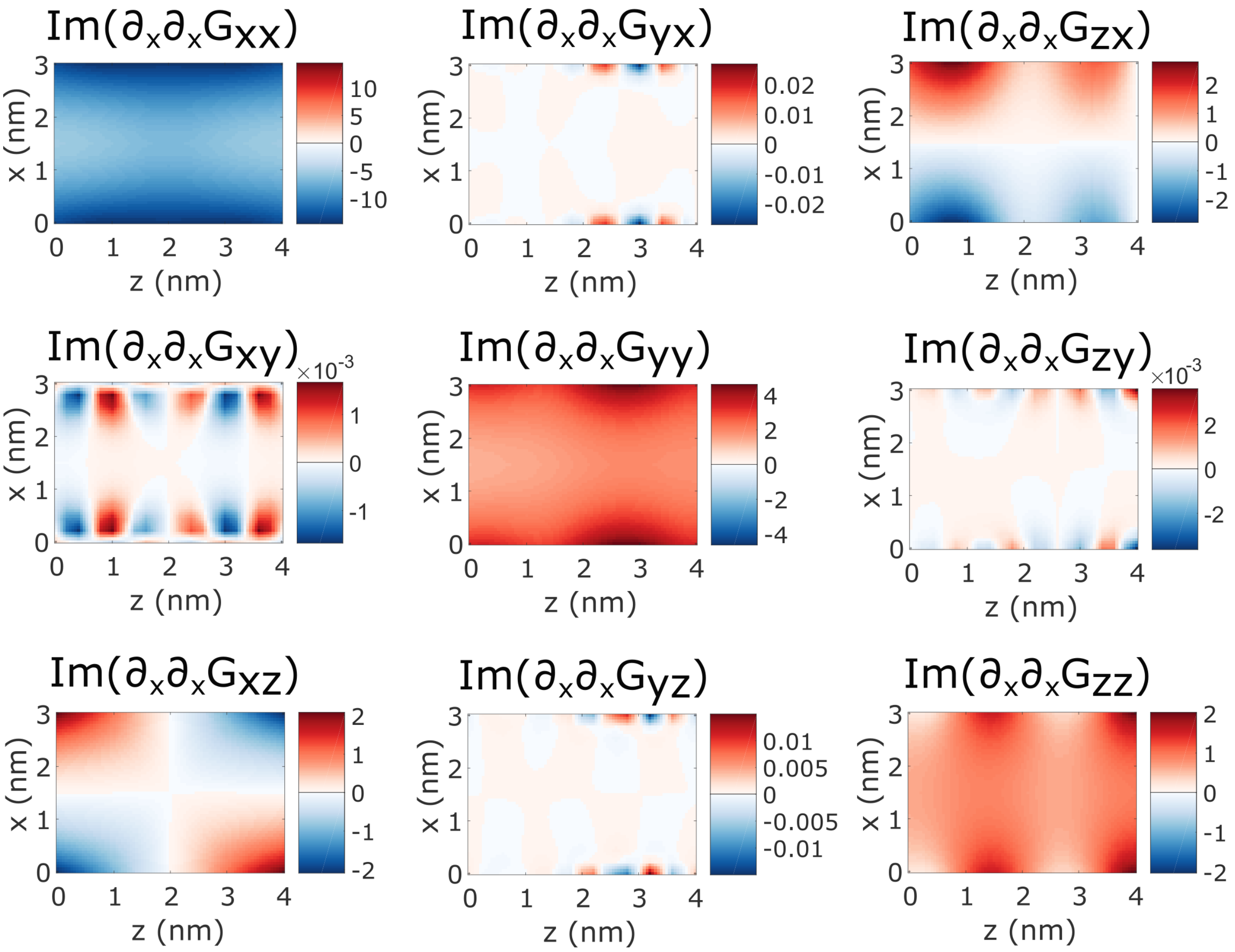

Figure S6. Green's tensor second derivative along x direction evaluated at the position of source (in nm^{-3}). Figure created in MATLAB R2019a (www.mathworks.com).

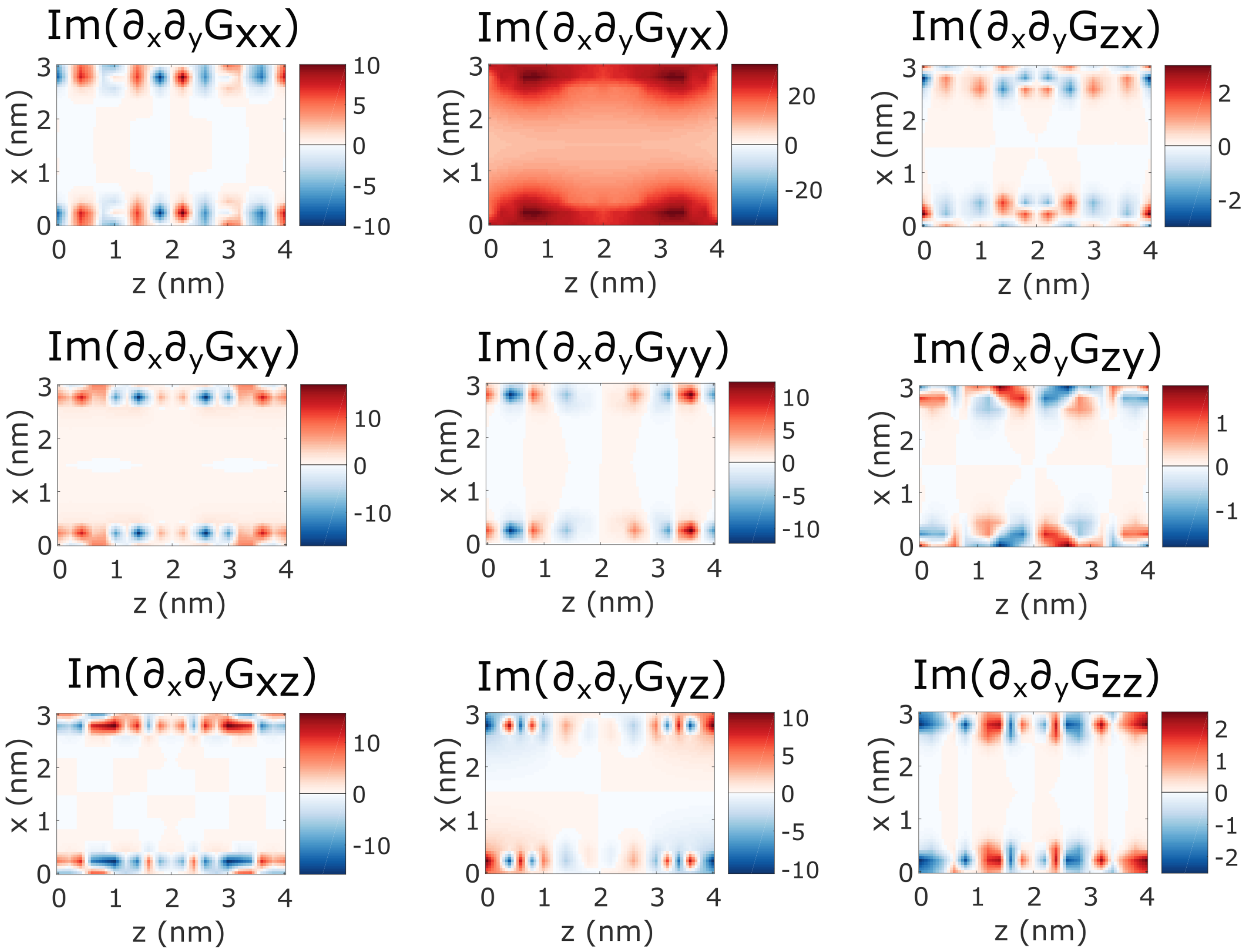

Figure S7. Green's tensor second derivative along x and along y direction evaluated at the position of source (in nm^{-3}). Figure created in MATLAB R2019a (www.mathworks.com).

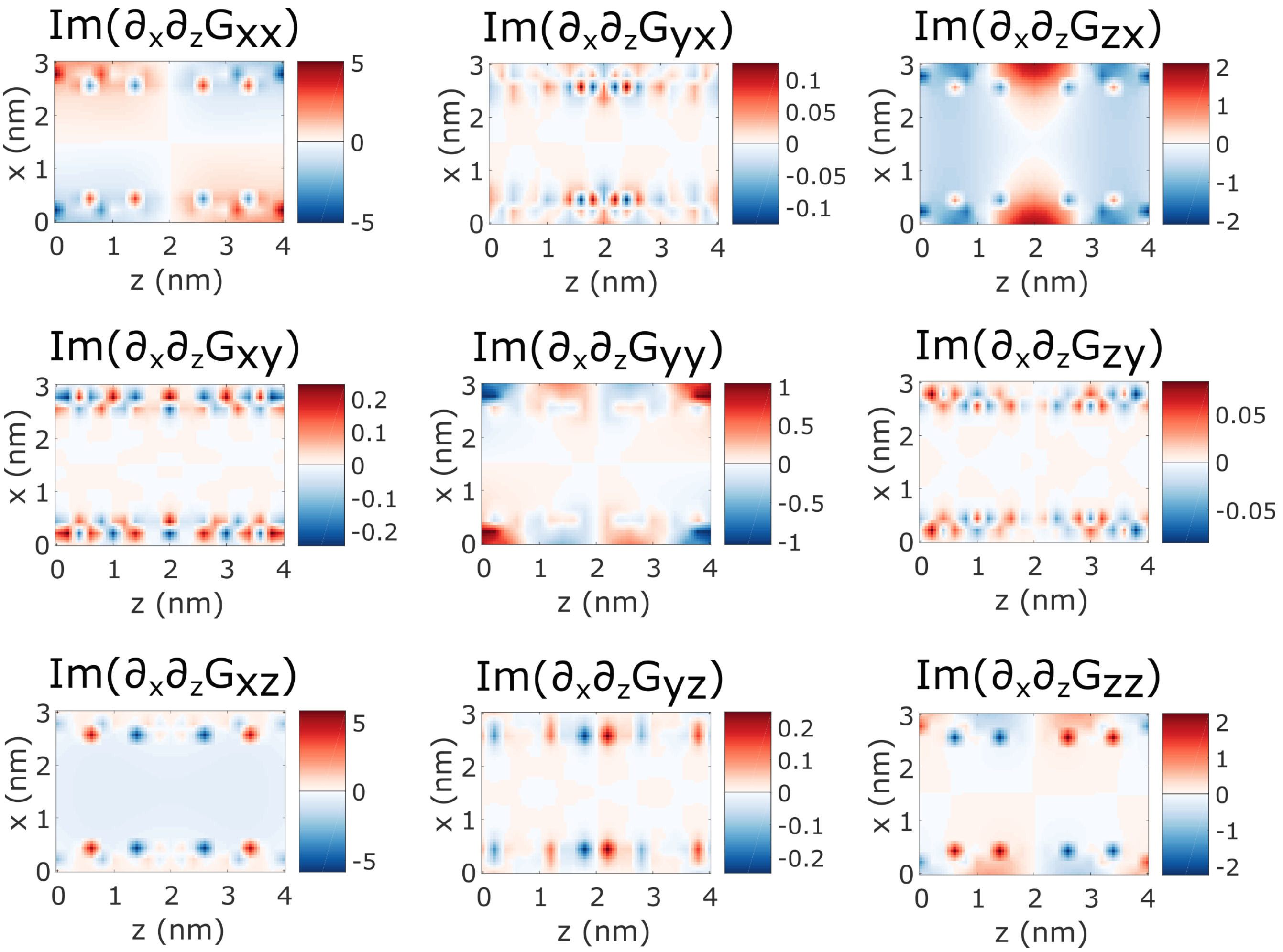

Figure S8. Green's tensor second derivative along x and along z direction evaluated at the position of source (in nm^{-3}).Figure created in MATLAB R2019a (www.mathworks.com).

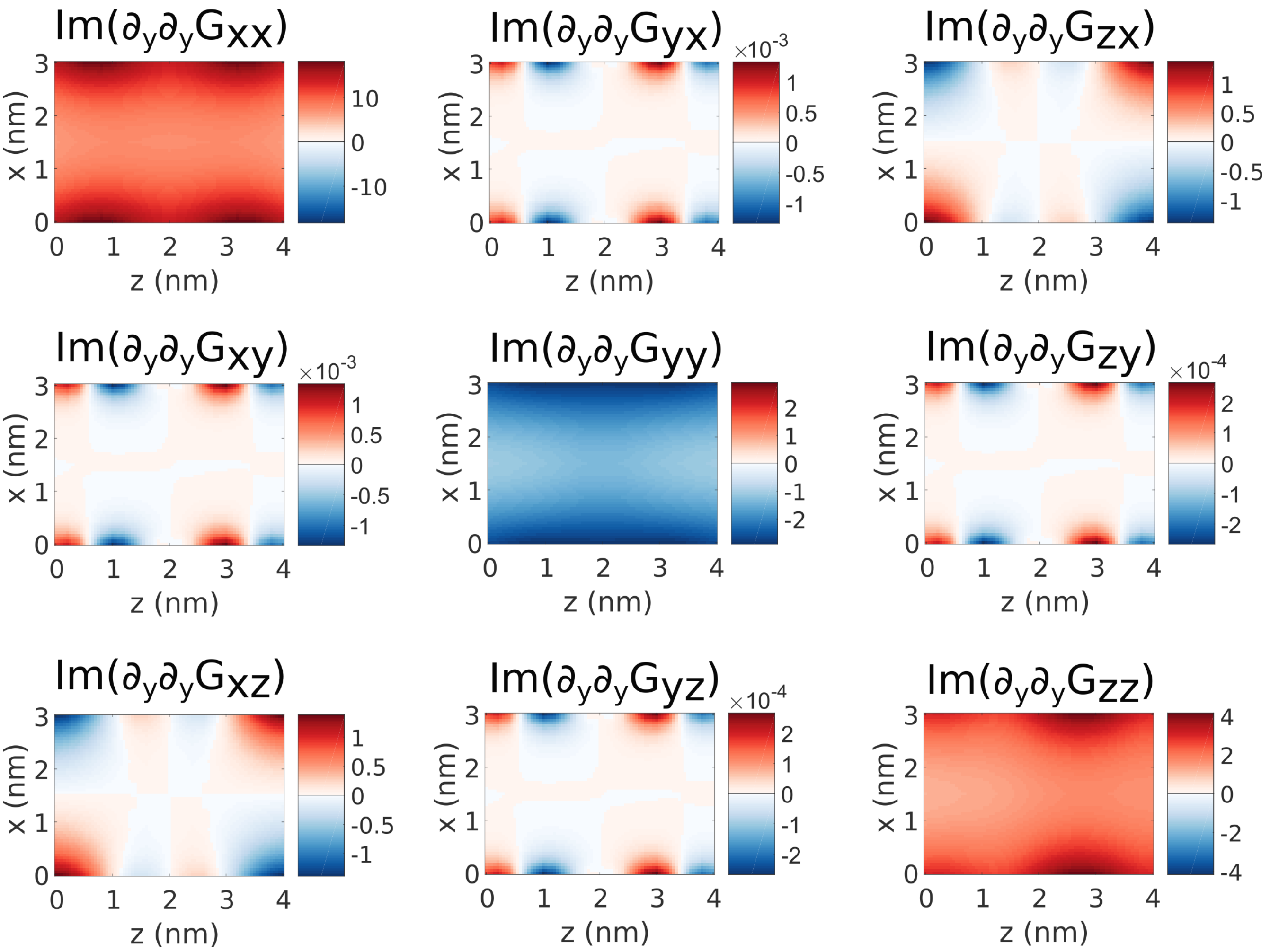

Figure S9. Green's tensor second derivative along y direction evaluated at the position of source (in nm^{-3}). Figure created in MATLAB R2019a (www.mathworks.com).

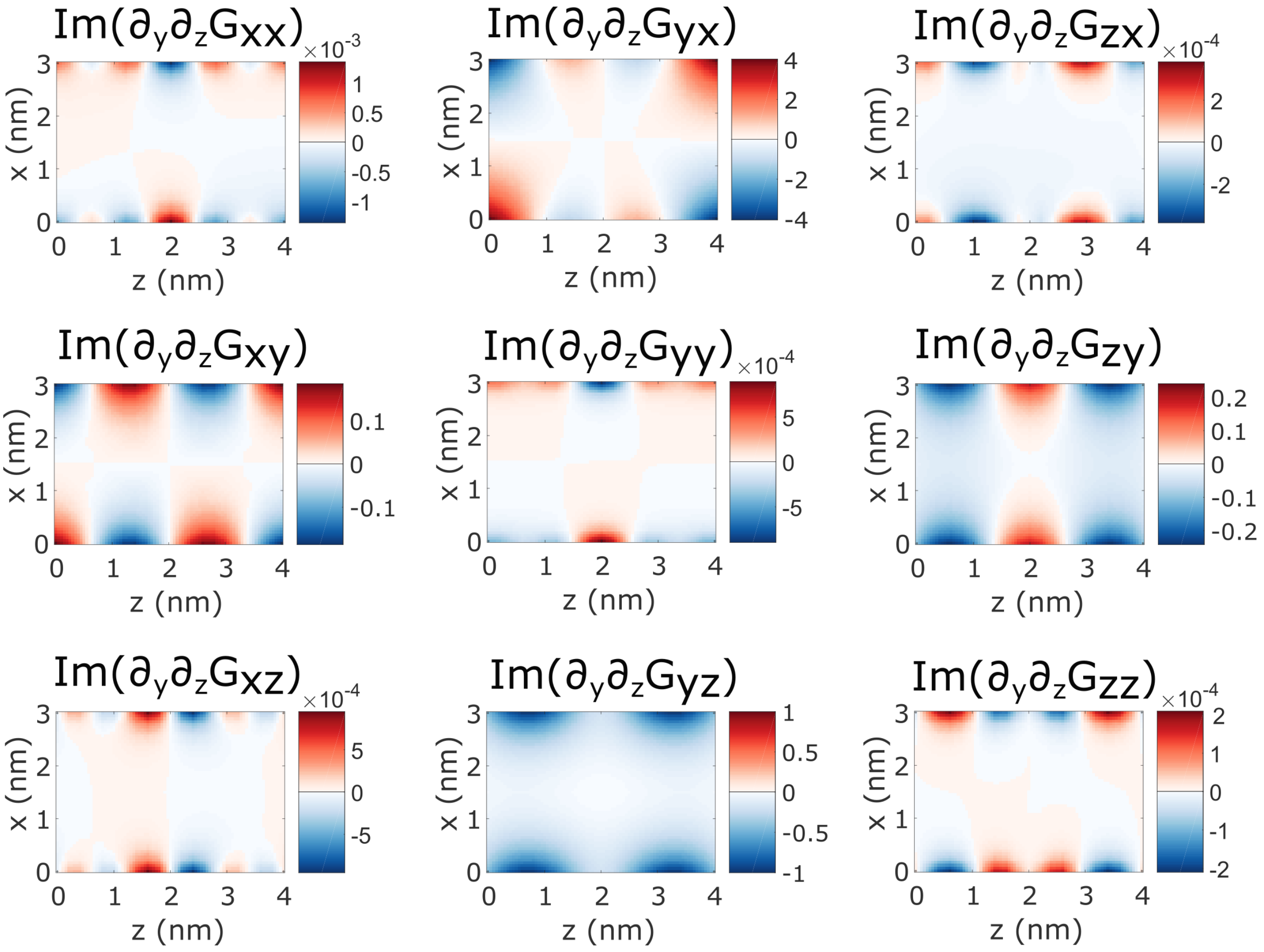

Figure S10. Green's tensor second derivative along y and along z direction evaluated at the position of source (in nm^{-3}). Figure created in MATLAB (version 2019Ra, https://www.mathworks.com/).

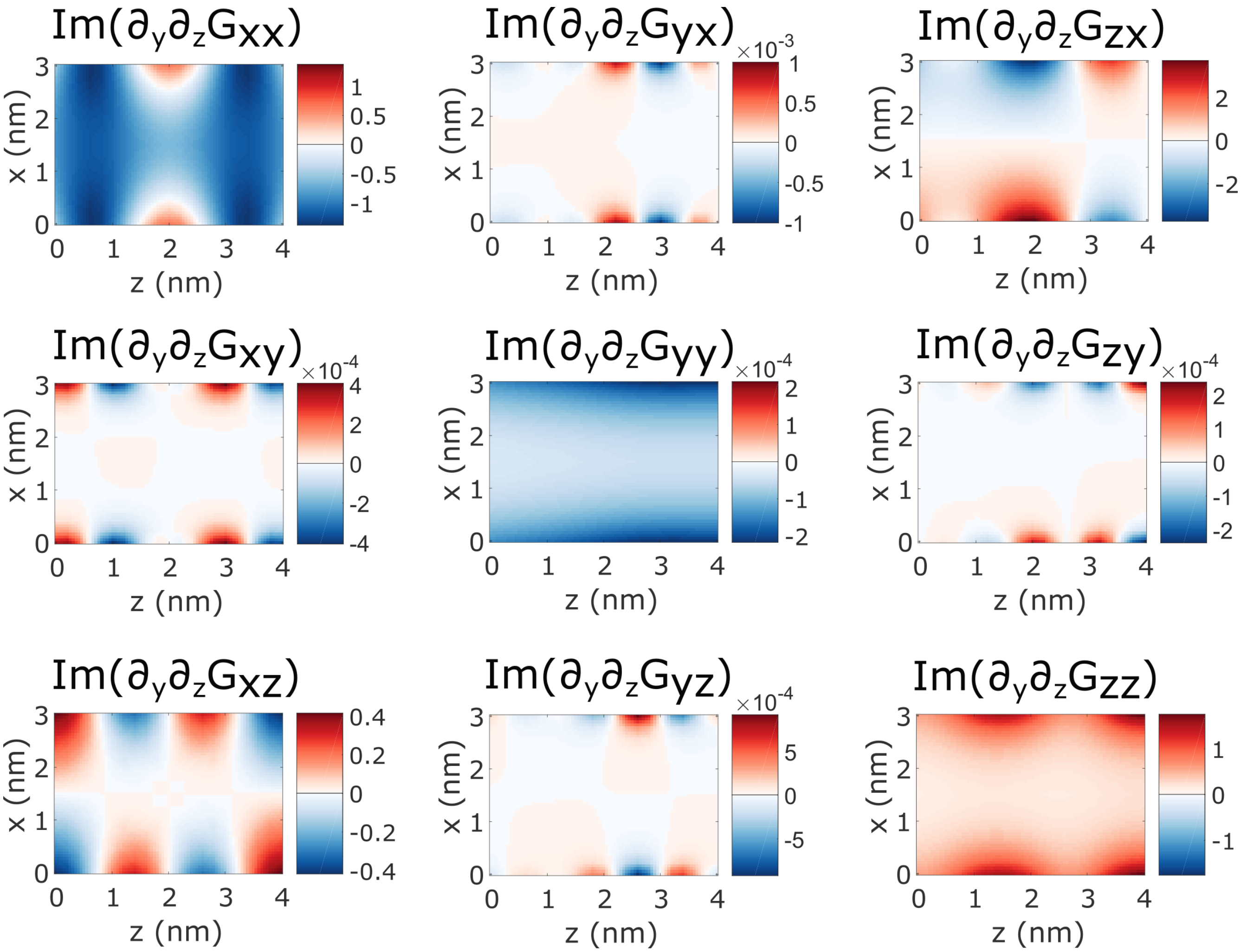

Figure S11. Green's tensor second derivative along z direction evaluated at the position of source (in nm^{-3}). Figure created in MATLAB R2019a (www.mathworks.com).
